# Microglia are involved in phagocytosis and extracellular digestion during Zika virus encephalitis in young adult immunodeficient mice

**DOI:** 10.1186/s12974-021-02221-z

**Published:** 2021-08-16

**Authors:** William Enlow, Maude Bordeleau, Jocelyne Piret, Fernando González Ibáñez, Olus Uyar, Marie-Christine Venable, Nathalie Goyette, Julie Carbonneau, Marie-Eve Tremblay, Guy Boivin

**Affiliations:** 1 Centre de Recherche en Infectiologie, Centre de Recherche du CHU de Québec-Université Laval, Quebec City, QC Canada; 2 Integrated Program in Neuroscience, McGill University, Montreal, QC Canada; 3 Neurosciences Axis, Centre de recherche du CHU de Québec–Université Laval, Quebec City, QC Canada; 4 Department of Molecular Medicine, Université Laval, Quebec City, QC Canada; 5 Division of Medical Sciences, University of Victoria, Victoria, BC Canada; 6 Department of Neurology and Neurosurgery, McGill University, Montreal, QC Canada; 7 Department of Biochemistry and Molecular Biology, The University of British Columbia, Vancouver, BC Canada

**Keywords:** Zika virus, Encephalitis, Microglia, Astrocytes, Neurons, Phagocytosis, Extracellular digestion, Neuroinflammation

## Abstract

**Background:**

Zika virus (ZIKV) has been associated with several neurological complications in adult patients.

**Methods:**

We used a mouse model deficient in TRIF and IPS-1 adaptor proteins, which are involved in type I interferon production, to study the role of microglia during brain infection by ZIKV. Young adult mice were infected intravenously with the contemporary ZIKV strain PRVABC59 (1 × 10^5^ PFUs/100 µL).

**Results:**

Infected mice did not present overt clinical signs of the disease nor body weight loss compared with noninfected animals. However, mice exhibited a viremia and a brain viral load that were maximal (1.3 × 10^5^ genome copies/mL and 9.8 × 10^7^ genome copies/g of brain) on days 3 and 7 post-infection (p.i.), respectively. Immunohistochemistry analysis showed that ZIKV antigens were distributed in several regions of the brain, especially the dorsal hippocampus. The number of Iba1^+^/TMEM119^+^ microglia remained similar in infected *versus* noninfected mice, but their cell body and arborization areas significantly increased in the *stratum radiatum* and *stratum lacunosum-moleculare* layers of the dorsal hippocampus *cornu ammoni* (CA)1, indicating a reactive state. Ultrastructural analyses also revealed that microglia displayed increased phagocytic activities and extracellular digestion of degraded elements during infection. Mice pharmacologically depleted in microglia with PLX5622 presented a higher brain viral load compared to untreated group (2.8 × 10^10^ *versus* 8.5 × 10^8^ genome copies/g of brain on day 10 p.i.) as well as an increased number of ZIKV antigens labeled with immunogold in the cytoplasm and endoplasmic reticulum of neurons and astrocytes indicating an enhanced viral replication. Furthermore, endosomes of astrocytes contained nanogold particles together with digested materials, suggesting a compensatory phagocytic activity upon microglial depletion.

**Conclusions:**

These results indicate that microglia are involved in the control of ZIKV replication and/or its elimination in the brain. After depletion of microglia, the removal of ZIKV-infected cells by phagocytosis could be partly compensated by astrocytes.

**Supplementary Information:**

The online version contains supplementary material available at 10.1186/s12974-021-02221-z.

## Background

Zika virus (ZIKV) is an arbovirus belonging to the *Flaviviridae* family. ZIKV is predominantly spread by *Aedes* mosquitoes and generally causes a mild and self-limiting febrile illness. However, during the 2013–2017 epidemic, an increasing number of neurological and peripheral nervous system complications related to infection with contemporary strains of ZIKV (Asian lineage) were reported in the South Pacific and Latin America. Epidemiological findings have established a relationship between ZIKV and congenital malformations including microcephaly [1–3]. Furthermore, ZIKV has been associated with Guillain-Barré syndrome as well as with other neurological disorders such as encephalitis, meningitis, encephalomyelitis, myelitis, and seizures in adult patients [4–7]. ZIKV has been shown to infect and replicate in mature neurons in cultured temporal lobe slices of human brains [8]. Viral RNA was reported to persist for up to 42 days in the cerebrospinal fluid (CSF) of young adult rhesus monkeys infected by ZIKV [9]. ZIKV RNA has been detected by RT-PCR in the CSF and brain of adult patients presenting with neurological disorders [10, 11]. Infectious virus was also isolated from CSF samples by culture on permissive cells [12]. Analysis of the CSF revealed lymphocytic pleocytosis, elevated protein levels, and normal glucose, suggesting a neuroinflammatory process [12, 13]. Magnetic resonance imaging showed brain abnormalities similar to those caused by other flaviviruses such as West Nile virus and Japanese encephalitis virus, but no specific findings could be associated with ZIKV [14]. These data suggest that ZIKV can invade the central nervous system (CNS) of adult individuals and promote neuroinflammation. However, the mechanisms underlying neurological disorders induced by ZIKV in the mature brain have not been completely elucidated.

In humans, ZIKV NS5 nonstructural protein induces the proteosomal degradation of interferon (IFN)-regulated transcriptional activator STAT2 and reduces type I IFN production [15]. This mechanism of immune evasion does not occur in mice, such that adult wild-type (WT) mice infected with ZIKV only develop a viremia without brain involvement. This limitation has required the administration of an anti-IFNAR (interferon alpha/beta receptor) antibody to WT mice [16] or the use of mice deficient in IFN receptors to study the pathogenesis of ZIKV infection in mature brains [17–19]. More recently, the neuropathogenesis of ZIKV was investigated in adult immunocompetent mice infected by intracranial inoculation of the virus [8, 20]. Viral RNAs are recognized by TLR3 (Toll-like receptor 3) and RIG-I (retinoic acid–inducible gene I/)/MDA-5 (melanoma differentiation–associated gene 5) leading to the production of type I IFNs. Our group has previously demonstrated that young adult mice deficient in TRIF (i.e., Toll-interleukin-1 receptor domain–containing adaptor inducing IFN-β) and IPS-1 (i.e., IFN-β promoter stimulator 1), the adaptor proteins of TLR3 and RIG-I/MDA-5, respectively, neither exhibited clinical signs of the disease nor mortality, when infected by the intravenous route with the contemporary ZIKV strain PRVABC59 [21]. Interestingly, these mice exhibited increased and sustained viral RNA loads in the serum, spleen, eyes, and brain compared with their WT counterparts. Although our TRIF^−/−^xIPS-1^−/−^ mice were deficient for the production of type I IFNs, these animals did not develop severe infection compared with IFNAR^−/−^ mice after ZIKV challenge [20]. Thus, TRIF^−/−^xIPS-1^−/−^ mice are a convenient model to study the pathogenesis of brain infection with a contemporary strain of ZIKV.

Microglia play an important role in neural development, functioning, and plasticity in the normal brain [22, 23]. Microglia also exert a key role in the surveillance and maintenance of tissue integrity through their dynamic ramifications, and they actively maintain homeostasis in the brain [24–26]. In cases of injury or invasion of pathogens in the CNS, microglia are the first line of defense and constitute the major phagocytic cells [27–29]. Following ZIKV infection in embryonic or young adult mouse models, microglia displayed morphological and functional changes including cell body enlargement, ramification remodeling, and increased phagocytic activities [16, 30, 31]. A recent study demonstrated that reactive resident microglia and infiltrating peripheral monocytes play a major role in lethal ZIKV encephalitis in immunocompetent mice infected intracranially with an African lineage strain [20].

As resident microglial cells constitute the first line of defense during viral infection of the brain parenchyma, we used our nonlethal young adult TRIF^−/−^xIPS-1^−/−^ mouse model infected intravenously with the contemporary ZIKV strain PRVABC59 to evaluate the physiological function of microglia in the almost complete absence of infiltrating peripheral monocytes. The effects of microglial depletion on brain infection with ZIKV were investigated in this mouse model using PLX5622, an inhibitor of the colony-stimulating factor 1 receptor tyrosine kinase activity required for microglial survival [32, 33]. Our results show that microglial cells display morphological characteristics of a reactive state during brain infection by ZIKV. Indeed, microglia show larger cell bodies and modified ramification areas. Ultrastructural analysis also revealed that microglia display phagocytic activities and extracellular digestion of degraded elements. Furthermore, we showed that microglia contribute to the control of ZIKV replication and/or its phagocytic elimination from the brain. Their depletion was associated with an increase in the number of ZIKV antigens stained by immunogold, especially in the cytoplasm and endoplasmic reticulum of neurons and astrocytes indicating an enhanced viral replication. Furthermore, the endosomes of astrocytes contained nanogold particles together with digested materials suggesting a compensatory role for these cells in phagocytosis.

## Materials and methods

### Animals

Five- to 6-week-old female TRIF^−/−^xIPS-1^−/−^ mice were used in this study. TRIF^−/−^xIPS-1^−/−^ mice were generated by Dr. Jean Gosselin (Université Laval, Quebec City, Quebec, Canada) and were maintained on a C57BL/6 background as previously described [21]. Animals were housed three to four per cage and acclimated to standard laboratory conditions.

### Infection of mice with ZIKV

TRIF^−/−^xIPS-1^−/−^ mice were infected with a low-passage, contemporary ZIKV strain (Asian lineage) isolated from a human serum specimen from Puerto Rico (PRVABC59) in 2015 [34]. An inoculum of 1 × 10^5^ plaque-forming units (PFUs) was administered intravenously in a volume of 100 µL of minimum essential medium supplemented with 2% fetal bovine serum (both from Life Technologies, Burlington, Ontario, Canada). Noninfected mice were used as controls. Some mice (*n* = 6 per group) were monitored for body weight change, clinical signs related to the infection (e.g., reduced mobility, hunched posture, partial or complete paralysis in hind or front limbs), and mortality for 14 days.

### Blood collection and preparation of brain homogenates

On days 0 (noninfected), 3, 7, 10, and 14 post-infection (p.i.), blood was collected from the mandibular vein of a subset of animals (*n* = 5–6 mice per time point) and centrifuged at 1500 × *g* for 10 min. Viral RNA loads were determined in serum as described below. Mice were then anesthetized by intraperitoneal injection of a mixture of 80 mg/kg ketamine hydrochloride (Bioniche Animal Health, Belleville, Ontario, Canada) and 10 mg/kg xylazine (Bimeda, Cambridge, Ontario, Canada) and sacrificed by intracardiac perfusion with cold 0.9% saline. Brains were harvested, weighed, and homogenized in 1 mL phosphate-buffered saline (PBS) containing protease (cOmplete) and phosphatase (PhosSTOP) inhibitor cocktails (both from Roche Applied Science, Laval, Quebec, Canada) using the Omni Tissue Homogenizer TH (OMNI International, Ottawa, Ontario, Canada). Brain homogenates were used for the determination of viral genomic RNA and cytokine/chemokine production as described below as well as IFN-α and -β mRNA levels (Supplementary methods: Quantification of interferon-α/-β mRNAs by reverse transcriptase droplet digital PCR).

### Determination of viral RNA load by reverse transcriptase droplet digital PCR

For serum, RNA was extracted from 100 µL of sample with the MagNA Pure LC DNA isolation kit (tissue; Roche Molecular System, Laval, Quebec, Canada) and recovered in 50 µL of elution buffer. Some samples were adjusted to 100 µL with sterile water, and the dilution factor was taken into account. Brain homogenates were centrifuged at 1500 × *g* for 10 min at 4 °C. Volumes of supernatants containing 0.01 g of brain were completed to 80 µL with H_2_O. RNA was then extracted after addition of 20 µL of enzymes using the same isolation kit and protocol as above and recovered in 50 µL of elution buffer. The determination of viral genomic RNA in serum and brain homogenates was done as described previously [35]. The droplet digital PCR (ddPCR) workflow and data analyses were performed with the One-Step reverse transcriptase ddPCR Advanced Supermix (Bio-Rad Laboratories, Mississauga, Ontario, Canada) according to the manufacturer’s instructions. Briefly, 20 µL of reaction mix was used for producing droplets with the QX200 droplet generator (Bio-Rad Laboratories). Droplet-partitioned samples were then transferred to a 96-well plate, sealed and cycled in a C1000 deep well thermocycler (Bio-Rad Laboratories). The cycled plate was then transferred and read in the FAM channel of the QX200 droplet reader (Bio-Rad Laboratories), and data analysis was performed using the QuantaSoft software (Version 1.7.4; Bio-Rad Laboratories).

### Cytokine/chemokine production

Brain homogenates were centrifuged at 10,000 × *g* for 10 min at 4 °C. A volume of 50 μL of supernatant was taken for the determination of cytokine and chemokine levels using a commercial multiplex mouse cytokine magnetic bead–based immunoassay (Bio-Plex Pro Mouse Cytokine 23-plex Assay; Bio-Rad Laboratories) according to the manufacturer’s instructions. Mean fluorescence intensity from all the bead combinations was analyzed using of the Bio-Plex system equipped with the Bio-Plex Manager Software v6.0 (Bio-Rad Laboratories).

### Tissue processing for light microscopy

A subset of animals (*n* = 5–6 mice per time point) was anesthetized with a mixture of 80 mg/kg ketamine hydrochloride and 10 mg/kg xylazine and sacrificed by intracardiac perfusion with PBS followed by 4% paraformaldehyde (PFA) on days 0 (noninfected), 3, 7, and 10 p.i.. Coronal brain sections. (30-µm thick) were obtained using a sliding microtome (Leica SM2010 R; Leica Biosystems Inc., Wetzlar, Germany) then placed in a cryoprotectant solution (30% glycerol and 30% ethylene glycol in PBS) and stored at – 20 °C until use.

### ZIKV distribution within the brain parenchyma by immunoperoxidase staining

Immunoperoxidase staining of ZIKV envelope antigens was performed as described previously [35]. Free-floating brain sections were quickly rinsed in PBS and washed in PBS 3 × for 10 min. Sections were incubated in 10 mM sodium citrate buffer containing 0.05% Tween 20 (at pH 6.0) in a water bath at 70 °C for 40 min, cooled down at room temperature (RT) for 5 min and washed in PBS 3 × for 10 min. Tissue sections were incubated in Dako dual endogenous block (Agilent Technologies, Santa Clara, CA, USA) for 10 min and washed in Tris-buffered saline (TBS) containing 0.05% Triton X-100 (TBS-T). Sections were incubated in blocking buffer (TBS-T containing 1% bovine serum albumin) for 20 min and then with a mouse IgG2a recombinant monoclonal antibody to Flavivirus group antigen (D1-4G2-4–15; Absolute Antibody, Oxford, UK) in blocking buffer (1:200) for 30 min and washed in TBS-T 3 × for 10 min. Sections were incubated with an anti-mouse Dako EnVision + horseradish peroxidase-labeled polymer (Agilent Technologies) for 30 min then washed in TBS 3 × for 10 min. Sections were recovered with Dako liquid diaminobenzidine (DAB) + substrate chromogen system (Agilent Technologies) and developed for 30 to 60 s. Sections were then washed in TBS 3 × for 10 min and mounted on glass slides. Tissue sections were dehydrated in ascending concentrations of ethanol and cleared in citriSolV solution (ThermoFisher Scientific, Waltham, MA, USA). Sections were rehydrated in descending concentrations of ethanol and counterstained with 0.1% Cresyl violet acetate (Millipore-Sigma, St. Louis, MO, USA) for 3 min. Slides were dehydrated again in ascending concentration of ethanol followed by citriSolV, then coverslipped with DPX mounting medium (Electron Microscopy Sciences, Hatfield, PA, USA).

Slides were examined at a magnification of 20 × using a NanoZoomer HT 2.0 slide scanner (Hamamatsu Photonics, Bridgewater, NJ, USA). In this analysis, Bregma − 1.35 to − 1.55 was selected to include the hippocampus region where the most intense immunostaining was detected [36]. The extent of ZIKV antigen immunostaining was determined by a scoring system (Supplementary Table 1). All the analyses were conducted blind to the experimental conditions.

### Immunofluorescence labeling of microglia and ZIKV antigens

Sections containing the dorsal hippocampus (selected Bregma − 1.31 to − 1.67; [36]) were selected for area, distribution, and morphological analyses of microglia on days 0 (noninfected), 7, and 10 p.i. by immunofluorescence microscopy. Tissue sections were quickly rinsed in PBS and washed in PBS 3 × for 10 min to remove the cryoprotectant solution. Antigen retrieval was performed by heating the sections with pre-heated 10 mM sodium citrate buffer containing 0.05% Tween 20 (at pH 6.0) at 70 °C for 10 min and then cooling down with citrate buffer at RT for 5 min. Sections were washed in PBS 5 × for 5 min, incubated in 0.1% NaBH_4_ in PBS for 30 min and washed in PBS 5 × for 5 min. Sections were then incubated in blocking buffer consisting of TBS containing 5% donkey serum, 0.5% gelatin (both from Millipore-Sigma) and 0.05% Triton X-100 at RT for 1 h. The blocking buffer was discarded, and sections were incubated with mouse anti-Flavivirus group antigen (D1-4G2-4–15; Absolute Antibody) (1:400), goat anti-Iba1 (ionized calcium-binding adapter 1) (MABN92; Millipore-Sigma) (1:1500), and rabbit anti-TMEM119 (transmembrane protein 119) (ab209064; Abcam, Cambridge, UK) (1:300) primary antibodies in blocking buffer at 4 °C overnight. Sections were put at RT for 15 min and washed in TBS-T. Sections were incubated with Alexa 488-conjugated donkey anti-mouse, Alexa 568-conjugated donkey anti-goat and Alexa 647-conjugated donkey anti-rabbit secondary antibodies (all from Life Technologies) in blocking buffer (1:300) at RT for 1.5 h and washed in TBS 5 × for 5 min. Sections were washed in phosphate buffer (PB) 3 × for 5 min, mounted on positively charged glass slides and dried for 2 h. Slides were then treated in 70% ethanol for 5 min, in Autofluorescence Eliminator Reagent (Millipore-Sigma) for 5 min and in 70% ethanol 3 × for 1 min to eliminate tissue autofluorescence and washed in PB. Slides were coverslipped with Fluoromount-G mounting medium (Southern Biotech, Birmingham, AL, USA) and stored at 4 °C until use.

Fluorescence images of the *stratum radiatum* (*st rad*) and the *stratum lacunosum-moleculare* (*st lac mol)* regions of the dorsal hippocampus *cornu ammoni* (CA)1 were captured at 10 × using a Axio Imager M2 epifluorescence microscope (Zeiss, Oberkochen, Germany) equipped with an AxioCam MRm camera and a 20 × Confocal Quorum WaveFX spinning disk confocal microscope (Quorum Technologies, Guelph, Ontario, Canada) equipped with a Hamamatsu ImageEM camera (Hamamatsu Corporation). Z-stack images acquired by confocal microscope were merged into a single-plane image using the Volocity 4 software (Perkin Elmer, Wellesley, MA, USA).

### Microglial population density, distribution, morphology, and co-localization with ZIKV antigens

All analyses were performed with the ImageJ software (National Institutes of Health, Bethesda, MD, USA) as previously described [37]. Briefly, for microglial density and spacing, the areas examined in the *st rad* and the *st lac mol* were delineated using the freehand tool in 10 × pictures (5–6 hippocampi/animal) and measured (µm^2^). The center of each individual Iba1^+^/TMEM119^+^ microglial cell body was marked with a dot by the paintbrush tool. Cell number and spatial coordinates were automatically recorded using the analyze particles function to determine the nearest neighbor distance (NND) using the NND plugin. Clusters comprising two or more microglial cells closer than 12 µm from one another were counted and averaged by animal. Density (cells number/area) and spacing index (mean NND^2^ × density) of microglial cells were calculated for every image and averaged by animal. To analyze morphology, a total of 17–21 microglial cells per animals were examined at 20 × . Only complete microglial cells in perfect focus were included in the analysis. For each Iba1^+^/TMEM119^+^ microglia, the soma area was determined by tracing the edge of the cell body with the freehand tool, while the arborization area was evaluated by connecting the most distal extremities of every process with the selection tool. A morphological index was calculated as the ratio of soma area to arborization area in order to evaluate the proportion of cell arborization [37, 38]. The shape descriptors were also determined (i.e., circularity, solidity, aspect ratio, and roundness). Circularity was calculated as 4πx(area/perimeter^2^), for which a value of 1.0 represents a perfect circle and around 0.0 an elongated shape. Solidity was calculated by dividing the cell area by the convex cell area meaning that a value close to 0.0 indicates a porous shape and close to 1.0 a convex shape. The aspect ratio was calculated by dividing the major axis by the minor axis of the cell; a value of 1.0 means similar minor and major axes, and higher values indicate that the cell is more elongated. Finally, roundness was calculated as 4 × area of microglia soma/(πx major axis^2^). During morphological analyses, the percentage of Iba1^+^/TMEM119^+^ microglial cells co-localizing with ZIKV antigen staining was calculated. In addition, the density of Iba1^+^/TMEM119^−^ infiltrating myeloid cells (cell number/area) and their ratio over Iba1^+^ cells was determined as previously reported [39]. All the analyses were conducted blind to the experimental conditions.

### Tissue processing for electron microscopy

On days 0 (noninfected) and 7 p.i. (which corresponds to the peak of brain viral load), a subset of animals (*n* = 4 mice per time point) was anesthetized with a mixture of 80 mg/kg ketamine hydrochloride and 10 mg/kg xylazine. Mice were blood-flushed by intracardiac perfusion with ~ 15 mL of ice-cold PBS and then with ~ 250 mL of a solution of 4% PFA/0.2% glutaraldehyde in 100 mM PB (pH 7.4). Brains were post-fixed in 4% PFA on ice for 2 h and washed in PBS 3 × for 10 min. Coronal sections of the brain (50-µm thick) were obtained using a vibratome (VT1200 S, Leica) and stored in a cryoprotectant solution at – 20 °C until use [40]. Sections containing the dorsal hippocampus (selected Bregma − 1.55; [36]) were used for immunostaining against ZIKV antigens. Sections were washed in PBS 3 × for 10 min. Sections were quenched with 0.3% H_2_O_2_ in PBS for 10 min and washed in PBS 3 × for 10 min. Sections were then permeabilized with 0.1% NaBH_4_ in PBS for 30 min and washed in PBS 3 × for 10 min. Sections were further incubated in blocking solution consisting of 5% normal goat serum, 1% bovine serum albumin, 0.05% Triton X-100 (all from Millipore-Sigma) in TBS at RT for 60 min. Sections were incubated with goat anti-mouse Fc receptor (Jackson ImmunoResearch, West Grove, PA) in TBS (1:12) for 30 min. Sections were then incubated in mouse anti-Flavivirus (Absolute Antibody) primary antibody (1:400) in blocking buffer at 4ºC overnight and washed in TBS-T 5 × for 5 min. Sections were incubated in goat anti-mouse 1.4 nm Nanogold-conjugated (Nanoprobes, Yaphank, NY, USA) secondary antibody (1:50) in TBS at 4 °C overnight and washed in TBS 5 × for 5 min and then in 3% sodium acetate solution 2 × for 5 min. Sections were finally revealed by the use of the silver enhancement kit (Nanoprobes) at RT for 1 min. Sections were quickly rinsed in 3% sodium acetate solution, further rinsed in PB 3 × for 5 min and washed in PBS 5 × for 3 min. Sections were incubated in a mixture containing equal volumes of 3% potassium ferrocyanide (BioShop, Burlington, Ontario, Canada) in 0.1 M PB and 4% aqueous osmium tetroxide (Electron Microscopy Sciences) at RT for 1 h and washed in milliQ water 5 × for 3 min. Sections were placed in 1% thiocarbohydrazide solution (Electron Microscopy Sciences) at RT for 20 min and rinsed in milliQ water 5 × for 3 min. Sections were then placed in 2% aqueous osmium tetroxide at RT for 30 min and washed in milliQ water 5 × for 3 min. Sections were then dehydrated in ascending concentrations of ethanol (2 × 35%, 1 × 50%, 1 × 70%, 1 × 80%, 1 × 90%, 3 × 100%) for 5 min followed by propylene oxide 3 × for 5 min. Sections were transferred in 100% Durcupan ACM resin (Electron Microscopy Sciences) for 24 h of infiltration at RT. Tissue sections were then embedded in a thin layer of resin between labeled ACLAR sheets (Electron Microscopy Sciences) and placed in an oven for 3 days of polymerization at 55 °C. After the polymerization, one of the ACLAR sheets was removed, a trapezoid shape of the region of interest was excised from resin-embedded tissue and glued on a resin block to process the embedded tissue for ultramicrotomy. Ultrathin (~ 70 nm) sections were cut with an ultramicrotome (Ultracut UC7 ultramicrotome, Leica Biosystems), collected on a silicon nitride chip (Electron Microscopy Sciences) and glued on specimen mounts (Electron Microscopy Sciences). Samples were imaged by array tomography at 5 nm (x, y) resolution for microglial ultrastructural analysis and 1 nm (x, y) resolution for cellular and subcellular localization analysis of nanogold labeling of ZIKV antigens using a Crossbeam 540 (GeminiSEM, Zeiss) field emission scanning electron microscope, with an acceleration voltage of 1.4 kV and current of 1.2 nA.

### Ultrastructural characterization of microglia

Ultrastructural analysis of microglia of ZIKV-infected (day 7 p.i.) and noninfected (day 0) mice in both layers of the dorsal hippocampus CA1 was performed blind to the experimental conditions using the ImageJ software (*st rad*: 32–33 microglia/time point, 6–10 microglia per animal; *st lac mol*: 32–36 microglia/time point, 7–10 microglia/animal). Microglia were recognized by their irregular nuclei with a heterogeneous chromatin pattern and a dark irregular cytoplasm, often containing long stretches of endoplasmic reticulum cisternae and lipidic inclusions (i.e. lipofuscin, lysosomes) [41]. Lysosomes, endosomes, lipidic inclusions, as well as altered endoplasmic reticulum, Golgi apparatus, and mitochondria within microglia were first analyzed quantitatively [42]. Lysosomes were identified by their dense heterogeneous contents enclosed by a single membrane [43, 44]. Mature lysosomes were differentiated from immature lysosomes by their contact with fusion endosomes and/or presence of lipofuscin granules (oval structure of finely granular composition with a typical fingerprint-like pattern) [43, 45]. Dilation of the endoplasmic reticulum and/or Golgi apparatus was noted when the distance between the two cisternal membranes was 50 nm or greater [46, 47]. Mitochondrial abnormalities that were quantified corresponded to elongated (length greater than 1 µm) [42], with hollow appearance, donut-shaped and abnormal [48] mitochondria. Microglial interactions with synaptic elements (i.e., presynaptic axon terminals or postsynaptic dendritic spines), myelinated axons, and degraded myelin of neurons were further identified. Presynaptic axon terminals were differentiated by their synaptic vesicles, while postsynaptic spines were in contact with a presynaptic axon terminal, often with a visible postsynaptic density at their junction [41]. Degraded myelin was recognized by the ballooning, swelling, or distancing of myelin sheaths [41]. Microglial interactions with other brain cell bodies (i.e., neurons and astrocytes) and blood vessels were quantified. Neurons were distinguished by their pale nuclei and pale cytoplasm, often with an apical dendrite and innervation from axon terminals. Astrocytic cells were recognized by their pale nucleus with a thin rim of heterochromatin as well as pale and irregular cytoplasm often containing intermediate filaments [41]. In the vicinity of microglia, the occurrence of degradation activities (e.g., myelin debris, extracellular digestion) was noted. Extracellular digestion or “exophagy” was identified as microglia-associated extracellular space pockets containing degraded elements or debris [49, 50].

### Effect of microglial depletion with PLX5622 on ZIKV infection of the brain

TRIF^−/−^xIPS-1^−/−^ mice received PLX5622 (Plexxikon Inc., Berkeley, CA, USA) formulated at a concentration of 1200 mg/kg in AIN-76A standard rodent diet (Research Diets Inc., New Brunswick, NJ, USA) or AIN-76A control diet ad libitum for 10 days. Blood and brain were collected in a subset of mice (*n* = 5 animals per group) the following day. The depletion of microglia was monitored by immunofluorescence staining against Iba1 in brain sections as described above. An evaluation of whether the treatment with PLX5622 could affect the number of total monocytes in the blood was also performed by flow cytometry analyses (Supplementary methods: Determination of blood monocyte levels by flow cytometry).

Groups of mice fed ad libitum with PLX5622 or control chow for 10 days were infected intravenously with ZIKV strain PRVABC59 (1 × 10^5^ PFUs in 100 µL). Feeding with the respective diets was continued for all the duration of the protocol. Mice (*n* = 3 per group) were monitored for body weight changes, clinical signs associated to the infection and mortality for 10 days as described above. Blood and brain of mice (*n* = 3 mice per group and per time point) were collected and processed for the determination of viral RNA load by reverse transcriptase ddPCR on days 0, 3, 7, and 10 p.i. as described above. Brain (*n* = 4 mice per group and per time point) was also harvested to evaluate the cellular and subcellular distribution of ZIKV antigens labeled with immunogold by scanning electron microscopy analysis on days 0 (noninfected) and 7 p.i. as described above.

### Cellular and subcellular distribution of ZIKV antigens labeled with immunogold

Pictures of all visible nanogold staining of ZIKV antigens in the *st rad* (36 pictures (7–13 per animal) for untreated and 47 pictures (9–14 per animal) for PLX5622-treated mice) and *st lac mol* (51 pictures (8–16 per animal) for untreated, and 59 pictures (9–22 per animal) for PLX5622-treated mice) layers of the dorsal hippocampus CA1 on day 7 after ZIKV challenge as well as representative pictures of noninfected animals were acquired. All images were taken within the tissue–resin border, which is the tissue part with the most intense staining. Distribution of nanogold particles was then analyzed qualitatively at the ultrastructural level by an observer blind to the experimental conditions. For each blinded picture, the brain cell type (i.e., neuron, astrocyte, oligodendrocyte, microglia, endothelial cell, pericyte, or other perivascular cells) containing nanogold particles as well as their localization within specific organelles (i.e., cytoplasm, endosome, endoplasmic reticulum, Golgi apparatus, plasma membrane, nuclear membrane, nucleus, and mitochondria membrane) were identified. Specificity of the staining distribution was evaluated by comparing noninfected *versus* infected mice on day 7 p.i. in both groups.

### Statistical analyses

All statistical analyses were performed using GraphPad Prism software program v8 (GraphPad Software, San Diego, CA, USA). The normal distribution of the data was assessed using a Shapiro-Wilks test and outliers were excluded by Grubb’s test, if data were normally distributed. A *P* value < 0.05 was considered as statistically significant. Viral load in serum and brain homogenates, cytokine/chemokine levels in brain homogenates, percentage of microglial cells co-localizing with ZIKV antigens, as well as density, distribution, and morphology of microglia were analyzed by a one-way analysis of variance (ANOVA) with Tukey’s multiple comparison test. Mean score of viral antigens staining and microglial clustering were analyzed using a nonparametric Kruskal–Wallis test for ordinal variable with Dunn’s multiple comparison test. Ultrastructural parameters of microglia were analyzed using a nonparametric Mann–Whitney comparison test.

## Results

### ZIKV is associated with a viremia and a brain viral load without causing mortality

**Fig. 1:**
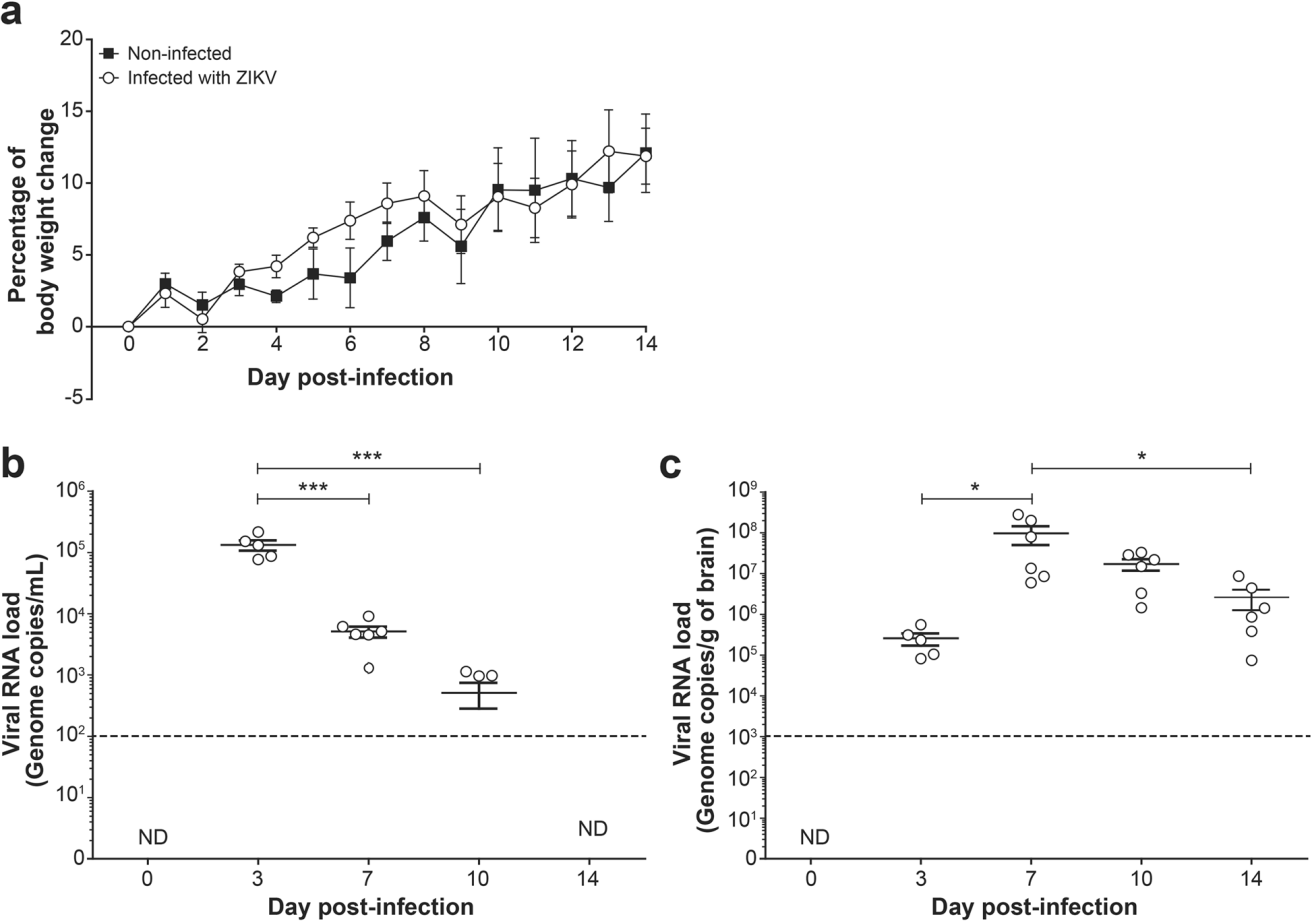
Body weight change and viral RNA load in serum and brain of young adult mice infected with ZIKV. TRIF^−/−^xIPS-1^−/−^ mice were infected intravenously with 1 × 10^5^ PFUs of ZIKV strain PRVABC59 in 100 µL volume. Noninfected mice were used as controls. **a** Body weight changes of ZIKV-infected (○) and noninfected (■) mice. Results represent the mean ± SEM of 6 mice per group. **b–c** Subsets of mice were sacrificed on days 0 (noninfected), 3, 7, 10, and 14 post-infection and viral RNA load was determined in serum (**b**) and brain homogenates (**c**) by reverse transcriptase ddPCR. Results are the mean ± SEM of 5–6 mice per time point. The dotted lines represent the limit of detection of the reverse transcriptase ddPCR assay. *ND*, not detected. Statistical analyses were performed using a one-way analysis of variance with Tukey’s multiple comparison test. Results that are statistically different between indicated groups are shown as follows: * *P* < 0.05; *** *P* < 0.001

Young adult TRIF^−/−^xIPS-1^−/−^ mice infected intravenously with 1 × 10^5^ PFUs of ZIKV strain PRVABC59 did not exhibit clinical signs of the disease, and all mice survived after infection. The body weight gain of noninfected and ZIKV-infected mice was similar throughout the monitoring period of 14 days (Fig. 1a). However, all mice challenged with ZIKV developed a viremia. The viral RNA load in serum was highest on day 3 (mean of 1.3 × 10^5^ genome copies/mL) and decreased thereafter to become undetectable by day 14 p.i. (Fig. 1b). Viral RNA was further detected in the brain on day 3 and reached a peak value (mean of 9.8 × 10^7^ genome copies/g of brain) on day 7 p.i. (Fig. 1c). The brain viral load decreased thereafter but was still detectable (mean of 2.6 × 10^6^ genome copies/g of brain) on day 14 p.i. Of note, we previously showed that age- and sex-matched WT C57BL/6 mice infected intravenously with the ZIKV strain PRVABC59 exhibited a viremia on day 1 that decreased below the limit of detection of the ddPCR assay on day 3 p.i. [21]. No viral load could be detected in the brain of WT mice after viral challenge. Thus, viral RNA load could be detected in the brain of young adult TRIF^−/−^xIPS-1^−/−^ mice from days 3 to 14 p.i. in the absence of clinical signs of infection.

### ZIKV increases the levels of selected cytokines and chemokines in the brain

The levels of cytokines and chemokines in brain homogenates were not significantly affected on day 3 p.i. compared with those measured on day 0 (Table 1). On day 7 p.i., the levels of some cytokines such as IL-1α (*P* < 0.05), IL-6 (*P* < 0.05), IL-9 (*P* < 0.01), IL-10 (*P* < 0.05), IL-12p70 (*P* < 0.01), and IFN-γ (*P* < 0.05) were significantly increased in ZIKV-infected mice compared with noninfected animals (day 0), whereas the increase was not significant for the levels of IL-12p40, G-CSF, and TNF-α. The levels of chemokines such as CCL2 (*P* < 0.05), CCL3 (*P* < 0.05), CCL4 (*P* < 0.05), CCL11 (*P* < 0.001), and CXCL1 (*P* < 0.05) were also significantly increased on day 7 p.i. in brain homogenates of ZIKV-infected *versus* noninfected animals. On the other hand, the levels of CCL5 were increased on day 7 compared with those measured on day 0, but the increase was statistically significant only on day 10 p.i.. The levels of IL-1β, IL-2, IL-3, IL-5, IL-13, and IL-17A were not affected after ZIKV challenge. As expected, the levels of IFN-α and -β mRNA transcripts in brain homogenates of TRIF^−/−^xIPS-1^−/−^ mice determined by reverse transcriptase droplet digital PCR were not affected after ZIKV infection (Supplementary Table 2) suggesting that no signaling pathways other than TLR3 and RIG-I/MDA-5 are involved in type I IFN production. In summary, ZIKV infection induced the production of selected cytokines and chemokines in young adult mouse brains.

**Table 1:** Cytokine and chemokine levels in the brain of mice infected with Zika virus

Cytokines/chemokines	Amounts (mean ± SEM) in pg/mL			
	Day 0	Day 3 post-infection	Day 7 post-infection	Day 10 post-infection
IL-1α	4.28 ± 0.34	3.93 ± 0.47	10.44 ± 2.44*	8.24 ± 1.13
IL-1β	0.94 ± 0.02	0.97 ± 0.03	1.29 ± 0.20	1.08 ± 0.06
IL-2	3.20 ± 0.25	3.20 ± 0.40	4.36 ± 0.68	3.65 ± 0.21
IL-3	0.12 ± 0.08	0.34 ± 0.16	0.46 ± 0.17	0.21 ± 0.10
IL-5	0.75 ± 0.12	1.21 ± 0.20	1.42 ± 0.31	0.99 ± 0.13
IL-6	0.95 ± 0.03	1.20 ± 0.13	10.65 ± 4.02*	1.54 ± 0.26
IL-9	5.55 ± 0.04	4.77 ± 0.36	8.50 ± 0.94**	4.92 ± 0.49
IL-10	2.37 ± 0.98	3.05 ± 1.44	19.10 ± 6.40*	13.78 ± 3.65
IL-12p40	138.91 ± 8.62	134.58 ± 14.62	2307.21 ± 1072.27	2223.27 ± 637.44
IL-12p70	42.19 ± 3.09	60.26 ± 4.92	81.10 ± 11.42**	43.06 ± 4.01
IL-13	109.35 ± 5.43	127.71 ± 10.94	124.58 ± 8.20	108.49 ± 5.62
IL-17A	2.29 ± 0.18	2.15 ± 0.30	2.76 ± 0.41	2.61 ± 0.24
G-CSF	4.64 ± 1.55	2.30 ± 0.82	264.34 ± 138.48	19.88 ± 8.10
IFN-γ	6.51 ± 0.76	6.73 ± 0.75	14.62 ± 3.37*	8.46 ± 0.86
TNF-α	57.43 ± 12.71	42.86 ± 3.50	102.20 ± 30.40	34.76 ± 1.79
CCL2	87.61 ± 3.78	79.74 ± 3.85	934.59 ± 387.08*	284.89 ± 62.11
CCL3	10.72 ± 0.30	12.57 ± 0.32	130.09 ± 58.52*	59.93 ± 15.94
CCL4	4.09 ± 0.42	4.65 ± 0.36	9.28 ± 2.00*	7.13 ± 1.72
CCL5	ND	ND	277.48 ± 102.87	567.68 ± 180.00**
CCL11	44.91 ± 3.26	44.24 ± 3.28	180.29 ± 39.51***	82.63 ± 9.50
CXCL1	15.79 ± 1.10	12.43 ± 0.81	51.12 ± 15.74*	26.66 ± 3.40

*ND*, not detected; *SEM*, standard error of the mean

Results are the mean ± SEM of 5–6 mice per time point

Statistical analyses were performed using a one-way analysis of variance with Tukey’s multiple comparison test. Results that are statistically different compared to day 0 (noninfected mice) are presented as follows: * *P* < 0.05; ** *P* < 0.01; *** *P* < 0.001 and are indicated in bold

### ZIKV antigens are more abundant in the dorsal hippocampus

**Fig. 2:**
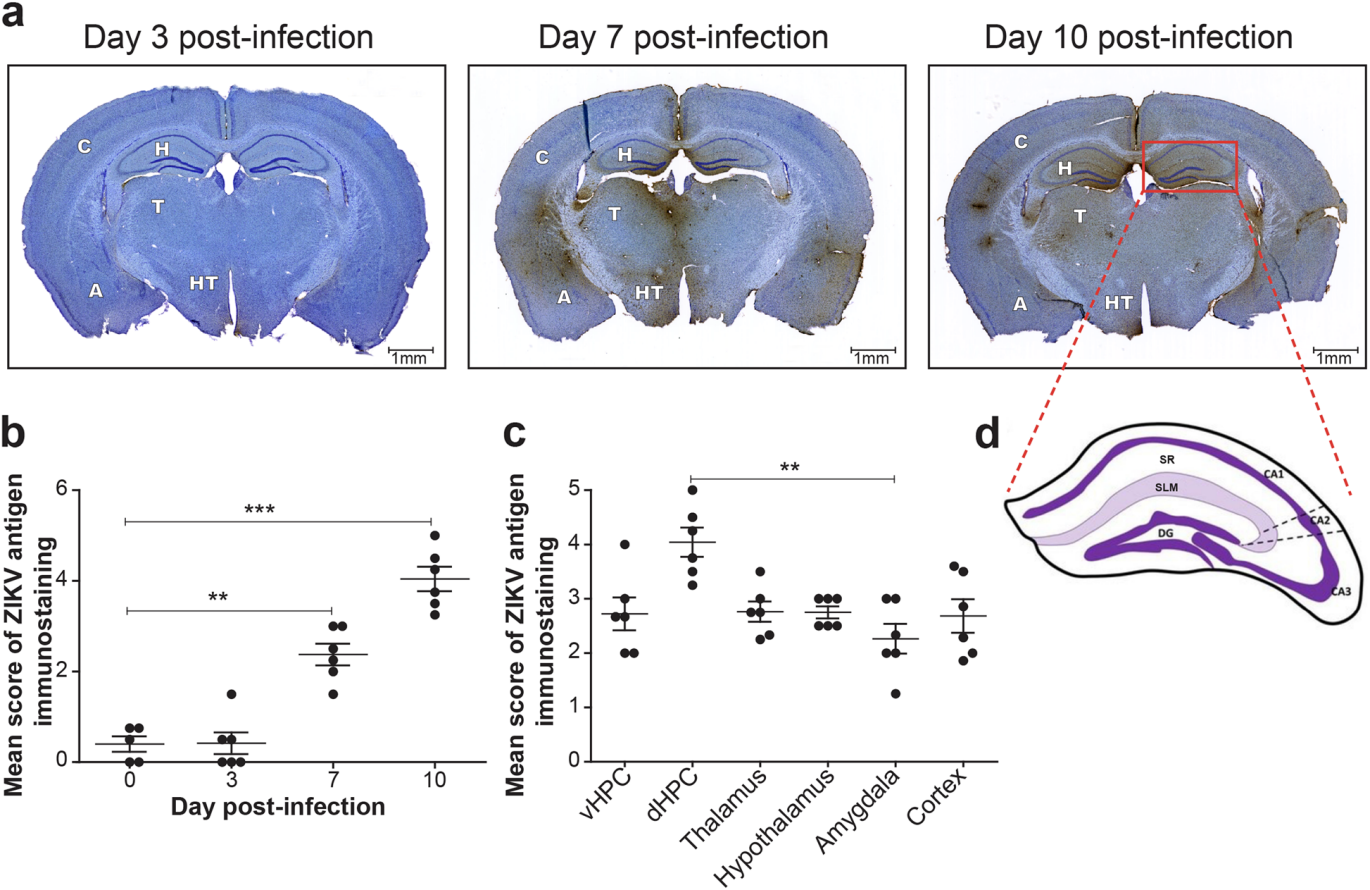
Immunoperoxidase staining of ZIKV antigens in young adult mouse brain. **a** Representative images illustrating the distribution of ZIKV envelope antigens stained by immunoperoxidase in brain sections on days 3, 7, and 10 post-infection. ZIKV-positive staining and cresyl violet-colored cells appear brown and purple-blue, respectively. Images were selected from slide scans obtained at a magnification of 20 × using a NanoZoomer HT 2.0 slide scanner. Scale bars on the picture are equivalent to 1 mm. **b** Mean score of ZIKV antigen immunostaining in the dorsal hippocampus at different times post-infection. Results represent the mean ± SEM of 5–6 mice per time point. **c** Mean score of ZIKV antigen immunostaining in selected brain regions on day 10 post-infection. Results represent the mean ± SEM of 6 mice per brain region. *H*, hippocampus; *vHPC*, ventral hippocampus; *dHPC*, dorsal hippocampus; *T*, thalamus; *HT*, hypothalamus; *A*, amygdala; *C*, cortex. Statistical analyses were performed using a nonparametric Kruskal–Wallis test for ordinal variable with Dunn’s multiple comparison test. Results that are statistically different between indicated groups are shown as follows: ** *P* < 0.01; *** *P* < 0.001. **d** Schematic representation of the dorsal hippocampus and the dentate gyrus (DG). The *cornu ammoni* 1 (CA1), CA2, and CA3 as well as the *stratum radiatum* (SR) and the *stratum lacunosum-moleculare* (SLM) layers are shown

To examine whether ZIKV infects specific regions of the brain, we performed immunoperoxidase staining of viral envelope antigens. ZIKV antigens were found in several regions of the brain including the ventral and dorsal hippocampi, the thalamus, the hypothalamus, the amygdala, and the cerebral cortex. Figure 2a shows that ZIKV antigens were detected in the dorsal hippocampus, the cortex, and the thalamus as early as on day 3 p.i. Immunostaining increased thereafter and was the most intense on day 10 p.i.. Figure 2b shows that the mean score of ZIKV antigen immunostaining in the dorsal hippocampus increased significantly on days 7 (*P* < 0.01) and 10 (*P* < 0.001) p.i. compared with day 0. The mean score of viral immunostaining was significantly higher in the dorsal hippocampus compared with the amygdala (*P* < 0.01) on day 10 p.i. (Fig. 2c). Thus, ZIKV mainly localized in the dorsal hippocampus among the young adult mouse brain, and its immunostaining was most intense on day 10 p.i.

### Microglia co-localize with ZIKV and increase their cell body and arborization areas

**Fig. 3:**
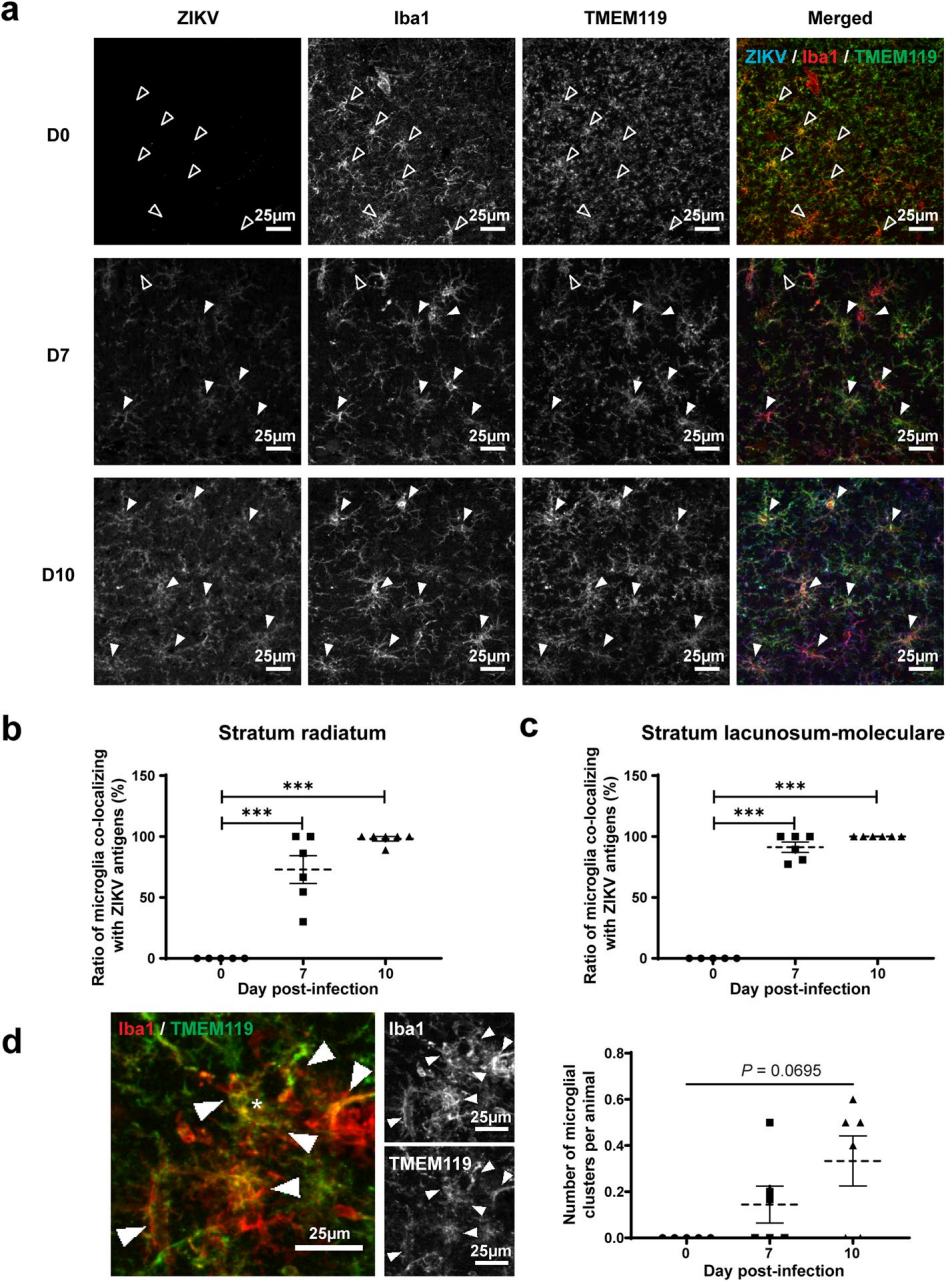
Co-localization of Iba1^+^/TMEM119^+^ microglia and ZIKV antigens in the dorsal hippocampus CA1. **a** Representative micrographs illustrating the localization of ZIKV antigens and Iba1^+^/TMEM119^+^ microglial cells after immunofluorescence labeling in the *st rad* on days 0 (noninfected), 7, and 10 post-infection. Filled and open arrowheads show microglia co-localizing with ZIKV or not, respectively. Scale bars on the picture are equivalent to 25 µm. **b–c** Percentage of Iba1^+^/TMEM119^+^ microglia co-localizing with ZIKV antigens in the *st rad* (**b**) and the *st lac mol* (**c**). **d** Representative micrographs showing a microglial cluster (indicated by an *) and number of microglial clusters per animal during ZIKV infection. Results are the mean ± SEM of 5–6 hippocampi per animal, for a total of 5–6 mice per time point. Statistical analysis was performed using a one-way ANOVA with Tukey’s multiple comparison test. Results that are statistically different between indicated groups are shown as follows: *** *P* < 0.001

In order to explore a potential interaction between microglia and ZIKV in the brain, we performed triple immunofluorescence staining of viral envelope antigens and of microglial cells with Iba1 and TMEM119 in the *st rad* and *st lac mol* layers of the dorsal hippocampus CA1 (see Fig. 2d for the anatomical localization). Figure 3a shows the distribution of Iba1^+^/TMEM119^+^ microglia and ZIKV antigens in the *st rad* of noninfected (day 0) and infected (days 7 and 10 p.i.) mice. Figures 3b and 3c show the percentage of Iba1^+^/TMEM119^+^ microglia that were found to co-localize with ZIKV antigens on days 7 and 10 p.i. in the *st rad* and *st lac mol*, respectively. By day 10 p.i., almost all Iba1^+^/TMEM119^+^ microglia were found to co-localize with ZIKV antigens in both layers of the dorsal hippocampus (*P* < 0.001 compared with day 0).

To identify possible functional alterations of microglia during ZIKV infection, we then conducted a detailed analysis of density, distribution, and morphology of Iba1^+^/TMEM119^+^ microglia in the *st rad* and *st lac mol* layers of the dorsal hippocampus CA1 using fluorescence microscopy. Microglial density, spacing index, and nearest neighbor distance were not affected during ZIKV infection compared with noninfected mice in both layers (Table 2). However, a nonsignificantly increased clustering of microglia, in which neighbor cells lose their territorial organization and become closer to one another, was observed in infected *versus* noninfected mice (Table 2; Fig. 3d). Despite the increased production of CCL2, the density of Iba1^+^/TMEM119^−^ myeloid cells and their ratio over Iba1^+^ cells were unaffected during ZIKV infection (Table 2). Thus, our mouse model allows the possibility to evaluate the role of microglia during brain infection with ZIKV without contributing effects of infiltrating peripheral monocytes.

**Table 2:** Density and distribution of Iba1^+^/TMEM119^+^ microglial cells and infiltrating Iba1^+^/TMEM119^−^ myeloid cells in both layers of the dorsal hippocampus CA1

Parameters	***Stratum radiatum***			***Stratum lacunosum-moleculare***		
	Day 0 Mean ± SEM	Day 7 p.i. Mean ± SEM	Day 10 p.i. Mean ± SEM	Day 0 Mean ± SEM	Day 7 p.i. Mean ± SEM	Day 10 p.i. Mean ± SEM
**Iba1**^**+**^**/TMEM119**^**+**^**microglial cells**						
Density (cells/mm^2^)	211.3 ± 4.8	206.2±20.4	218.0 ± 13.7	232.3 ± 11.0	220.9 ± 26.3	275.5 ± 23.8
Spacing index (a.u.)	0.474 ± 0.007	0.453±0.013	0.480 ± 0.018	0.484 ± 0.016	0.477 ± 0.014	0.497 ± 0.010
NND (μm)	47.75 ± 0.66	48.14±1.94	48.00 ± 1.49	45.80 ± 2.08	47.79 ± 3.04	43.97 ± 1.62
Number of clusters per animal	0.000 ± 0.000	0.150±0.081	0.333 ± 0.109	0.000 ± 0.000	0.150 ± 0.081	0.333 ± 0.109
**Infiltrating Iba1**^**+**^**/TMEM119**^**-**^**myeloid cells**						
Density (cells/mm^2^)	0.6393 ± 0.5245	0.1873 ± 0.1186	1.030 ± 0.664	3.510 ± 1.469	2.454 ± 0.786	4.843 ± 1.808
Ratio over Iba1^+^ cells (%)	0.2817 ± 0.2086	0.0940 ± 0.0604	0.4300 ± 0.2764	1.288 ± 0.510	1.102 ± 0.330	1.672 ± 0.458

*a.u.*, arbitrary unit; *NND*, nearest neighbor distance; *p.i.*, post-infection; *SEM*, standard error of the mean

Results are the mean ± SEM of 5–6 hippocampi per animal for a total of 5–6 mice per time point

Statistical analyses were performed using a one-way analysis of variance with Tukey’s multiple comparisons test

**Fig. 4:**
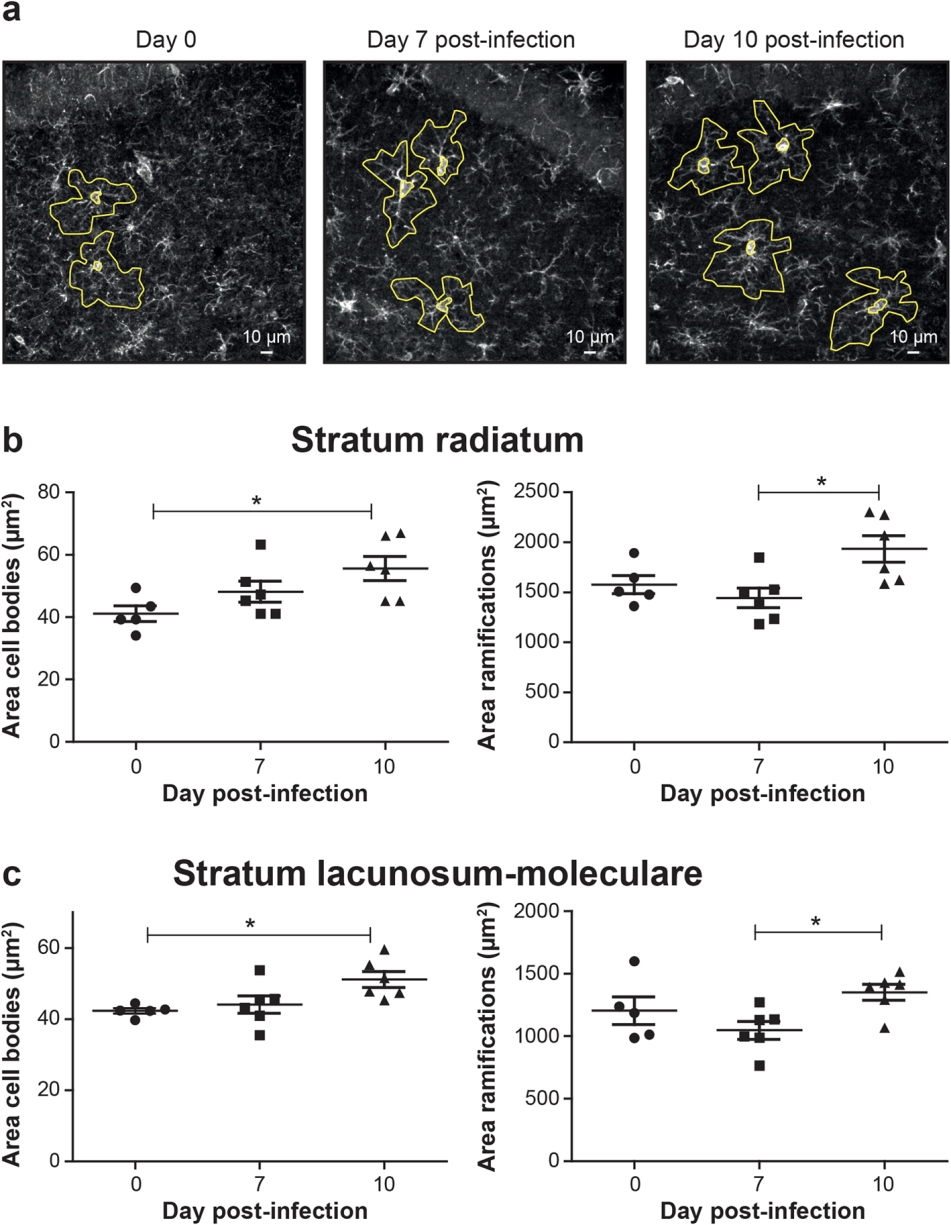
Morphology of Iba1^+^/TMEM119^+^ microglial cells in the dorsal hippocampus CA1 after ZIKV challenge. **a** Representative micrographs with examples of tracing around Iba1^+^/TMEM119^+^ microglia (using the Iba1^+^ channel) in the *st rad* on day 0 (noninfected), 7, and 10 post-infection that were used to calculate the areas of cell bodies and arborizations. Scale bars on the picture are equivalent to 10 µm. **b–c** Areas of cell bodies (left) and areas of ramifications (right) of Iba1^+^/TMEM119^+^ microglia in the *st rad* (**b**) and *st lac mol* (**c**). Results are the mean ± SEM of 17 to 21 microglial cells per animal for a total of 5–6 mice per time point. Statistical analysis was performed using a one-way ANOVA with Tukey’s multiple comparison test. Statistically different results between indicated groups are presented as follows: * *P* < 0.05

Furthermore, changes in microglial morphology were observed for both cell bodies and process arborization. Figure 4a shows examples of tracing around Iba1^+^/TMEM119^+^ microglia (using the Iba1^+^ channel) on days 0, 7, and 10 p.i. that was used to calculate the areas of cell bodies and arborization processes. On day 10 p.i., microglial cell body area was significantly increased compared with noninfected mice (*P* < 0.05), whereas their arborization area was significantly increased compared with day 7 p.i. (*P* < 0.05) in the *st rad* (Table 3; Fig. 4b) and the *st lac mol* (Table 3; Fig. 4c). However, the morphological index, calculated as the ratio between the soma and arbor areas, was not affected throughout the infection in both layers of the dorsal hippocampus CA1 (Table 3). No changes in the circularity, solidity, aspect ratio, and roundness of microglial cell body and arborization areas were observed in the *st rad*. In contrast, the shape descriptor analysis identified a significant decrease in the solidity of microglial arborization areas (*P* < 0.05) in the *st lac mol* of ZIKV-infected compared with noninfected mice on day 10 p.i.. In the *st lac mol*, the aspect ratio of microglial arborization areas was also significantly decreased (*P* < 0.05), while their roundness was significantly increased (*P* < 0.05) on day 10 compared to day 7 p.i. suggesting that microglial arborization area, corresponding to their surveyed territory, became rounder between days 7 and 10 p.i.. These changes in microglial cells suggest functional modifications in their interventions with parenchymal elements (such as neurons and other glial cells) and the vasculature in the brain of young adult mice infected with ZIKV.

**Table 3:** Morphological parameters of Iba1^+^/TMEM119^+^ microglia in both layers of the dorsal hippocampus CA1

Parameters	***Stratum radiatum***			***Stratum lacunosum-moleculare***		
	Day 0 Mean ± SEM	Day 7 p.i. Mean ± SEM	Day 10 p.i. Mean ± SEM	Day 0 Mean ± SEM	Day 7 p.i. Mean ± SEM	Day 10 p.i. Mean ± SEM
Cell area (μm^2^)	239.6 ± 19.0	250.3 ± 15.4	247.9 ± 12.9	146.8 ± 10.7	160.0 ± 93.5	150.2 ± 10.9
Soma area (μm^2^)	41.08 ± 2.55	48.18 ± 3.40	55.57 ± 3.89*	42.30 ± 0.76	44.06 ± 2.47	51.12 ± 2.22*
Soma perimeter (μm)	25.18 ± 0.92	27.74 ± 1.29	29.62 ± 1.15 (*P* = 0.0574)	25.35 ± 0.26	26.16 ± 0.92	28.36 ± 0.68*
Arbor area (μm^2^)	1576.2 ± 90.5	1443.2 ± 98.1	**1931.5 ± 132.2**^**#**^	1204.2 ± 110.0	1046.9 ± 70.8	**1351.6 ± 63.8**^**#**^
Arbor perimeter (μm)	186.0 ± 8.5	187.4 ± 11.1	**227.0 ± 11.8 (*P*= 0.0505)**^**#**^	163.4 ± 12.2	158.0 ± 7.7	185.2 ± 4.6
Morphological index (a.u.)	0.028 ± 0.001	0.036 ± 0.001	0.031 ± 0.003	0.041 ± 0.003	0.046 ± 0.002	0.041 ± 0.002
Circularity soma (a.u.)	0.814 ± 0.009	0.794 ± 0.019	0.794 ± 0.010	0.830 ± 0.008	0.810 ± 0.012	0.800 ± 0.011
Solidity soma (a.u.)	0.904 ± 0.005	0.902 ± 0.006	0.899 ± 0.004	0.916 ± 0.003	0.914 ± 0.005	0.914 ± 0.005
Aspect ratio soma (a.u.)	1.653 ± 0.027	1.712 ± 0.053	1.638 ± 0.045	1.611 ± 0.049	1.725 ± 0.057	1.757 ± 0.054
Roundness soma (a.u.)	0.651 ± 0.013	0.626 ± 0.015	0.649 ± 0.015	0.666 ± 0.013	0.620 ± 0.017	0.621 ± 0.013
Circularity arbor (a.u.)	0.581 ± 0.015	0.538 ± 0.024	0.487 ± 0.035	0.572 ± 0.031	0.542 ± 0.017	0.509 ± 0.014
Solidity arbor (a.u.)	0.847 ± 0.007	0.827 ± 0.012	0.804 ± 0.018	0.847 ± 0.013	0.828 ± 0.007	0.810 ± 0.008*
Aspect ratio arbor (a.u.)	1.498 ± 0.047	1.594 ± 0.031	1.478 ± 0.044	1.641 ± 0.047	1.757 ± 0.037	**1.567 ± 0.051**^**#**^
Roundness arbor (a.u.)	0.693 ± 0.020	0.657 ± 0.010	0.706 ± 0.016	0.643 ± 0.017	0.612 ± 0.008	**0.670 ± 0.021**^**#**^

*a.u.*, arbitrary units; *p.i*., post-infection; *SEM*, standard error of the mean

Results are the mean ± SEM of 17–21 microglia per animal for a total of 5–6 mice per time point

Statistical analyses were performed using a one-way analysis of variance with Tukey’s multiple comparison test. * *P* < 0.05 day 10 compared with day 0 (noninfected mice); ^#^ *P* < 0.05 day 10 compared with day 7 p.i.. Both are indicated in bold

### ZIKV increases microglial lysosome number and induces microglia-associated extracellular digestion

In order to assess the effect of ZIKV infection on microglial function, we analyzed ultrastructural changes in the organelles of microglial cells and their interactions with their direct environment in the *st rad* and *st lac mol* of mice on day 7 p.i. compared with noninfected animals using scanning electron microscopy. More precisely, for each microglia, we quantified the number of organelles involved in the phagolysosomal pathway (immature and mature lysosomes, endosomes with or without content and lipidic inclusions) and the number of altered organelles that serve as markers of cellular stress (dilated cisternae of endoplasmic reticulum and Golgi apparatus as well as elongated, with hollow appearance, donut-shaped and abnormal mitochondria). We also evaluated the number of contacts of microglial cells with their microenvironment, particularly with neurons (presynaptic axon terminals, postsynaptic dendritic spines), myelin (myelinated axons and degraded myelin), neuronal cell bodies, astrocytic cell bodies, and blood vessels. In the vicinity of microglia, we examined the presence of myelin debris, extracellular space pockets containing degraded elements or debris indicative of extracellular digestion or “exophagy”.

**Fig. 5:**
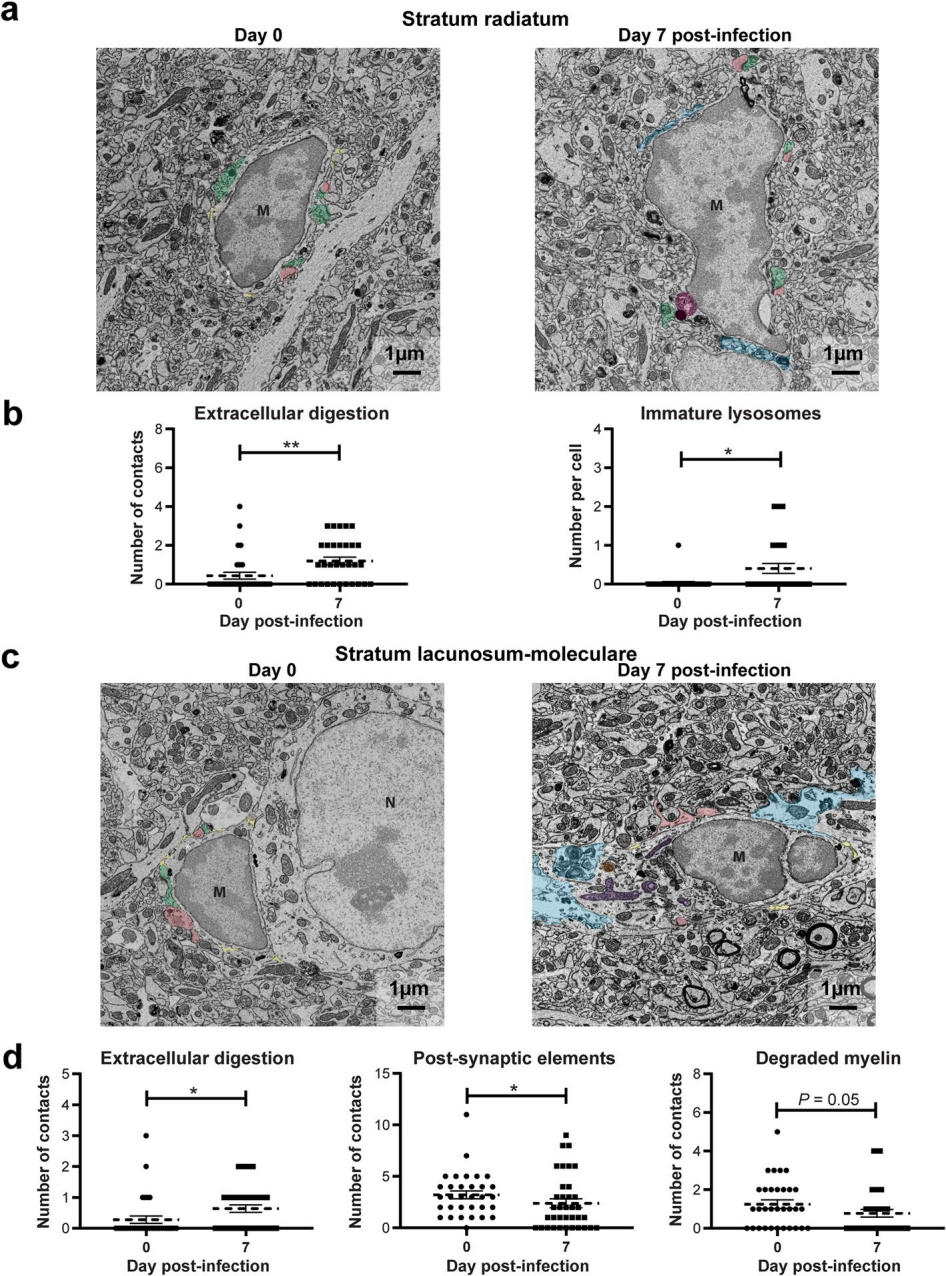
Ultrastructural analysis of the dorsal hippocampus CA1 of mice infected with ZIKV. **a** Example pictures of microglia in the *stratum radiatum* of noninfected (day 0) and infected mice (day 7 post-infection) highlighting changes in **b** extracellular digestion and the number of immature lysosomes per cell during ZIKV infection. Results are the mean ± SEM of 32–33 microglia per time point (6–10 microglia per animal for a total of 4 mice per time point). **c** Example pictures of microglia in the *stratum lacunosum-moleculare* of noninfected (day 0) and infected mice (day 7 post-infection) showing changes of **d** extracellular digestion and contacts with postsynaptic elements and degraded myelin per cell during ZIKV infection. Results are the mean ± SEM of 32–36 microglia per time point (7–10 microglia per animal for a total of 4 mice per time point). On the example pictures, cell types are identified by a capital letter; *M*, microglia; *N*, neuron. Structural elements on the example pictures are identified by a mix of pseudo-coloring and text annotation; presynaptic elements (green), postsynaptic elements (red), extracellular space (yellow), extracellular digestion (light blue), immature lysosomes (pink), mature lysosomes (orange), abnormal mitochondria (purple), and dilated endoplasmic reticulum (white asterisk). Scale bars on the picture are equivalent to 1 µm. Statistical analyses were performed using a nonparametric Mann–Whitney comparison test. Results that are statistically different between indicated groups are shown as follows: * *P* < 0.05; ** *P* < 0.01

In the *st rad*, the number of immature lysosomes and the overall number of lysosomes within microglia were significantly higher on day 7 p.i. compared with those on day 0 (*P* < 0.05), whereas no significant differences were seen for the other organelles (Table 4; Fig. 5a,b). Moreover, microglia-associated extracellular digestion was significantly higher on day 7 p.i. compared with that on day 0 (*P* < 0.01; Table 4; Fig. 5b). Microglial interactions with synaptic elements, myelin, neurons, astrocytes, blood vessels, as well as myelin debris and extracellular space pockets remained unchanged after infection. These results suggest an overall increase in microglial phagolysosomal activities in the *st rad* of the dorsal hippocampus CA1.

**Table 4:** Microglial ultrastructure in both layers of the dorsal hippocampus CA1

Parameters		Number per cell (mean ± SEM)			
		***Stratum radiatum***		***Stratum lacunosum-moleculare***	
		Day 0	Day 7 p.i.	Day 0	Day 7 p.i.
**Organelles of phagolysosomal pathway within microglia**					
Lysosomes	Immatures	0.031 ± 0.031	0.406 ± 0.126*	0.313 ± 0.123	0.306 ± 0.087
	Matures	0.094 ± 0.052	0.250 ± 0.078	0.344 ± 0.124	0.333 ± 0.089
	Total	0.125 ± 0.059	0.656 ± 0.159*	0.656 ± 0.159	0.639 ± 0.139
Endosomes	Empty	0.094 ± 0.052	0.188 ± 0.105	0.063 ± 0.043	0.222 ± 0.081
	With content	0.531 ± 0.180	0.281 ± 0.112	0.719 ± 0.175	0.472 ± 0.116
	Total	0.625 ± 0.178	0.469 ± 0.201	0.781 ± 0.189	0.694 ± 0.178
Lipidic inclusions		0.156 ± 0.079	0.250 ± 0.089	0.250 ± 0.100	0.417 ± 0.108
**Altered organelles within microglia**					
ER/Golgi	Dilation	0.875 ± 0.059	0.781 ± 0.074	0.906 ± 0.052	0.944 ± 0.039
Mitochondria	Elongated	0.188 ± 0.095	0.438 ± 0.134	0.594 ± 0.173	0.417 ± 0.128
	Holy	0.094 ± 0.052	0.094 ± 0.069	0.094 ± 0.052	0.139 ± 0.058
	Donut-shaped	0.031 ± 0.031	0 ± 0	0.031 ± 0.031	0.055 ± 0.039
	Abnormal	0.281 ± 0.103	0.375 ± 0.117	0.688 ± 0.182	0.611 ± 0.175
	Total (normal + altered)	2.188 ± 0.319	3.531 ± 0.741	3.531 ± 0.552	3.833 ± 0.531
**Contacts of microglia with microenvironment**					
Synaptic elements (neurons)	Pre-synaptic	4.406 ± 0.638	3.375 ± 0.451	5.813 ± 0.595	4.944 ± 0.569
	Post-synaptic	1.875 ± 0.361	2.094 ± 0.352	3.219 ± 0.380	2.389 ± 0.439*
Myelin (neurons)	Myelinated axons	0.344 ± 0.115	0.313 ± 0.138	0.625 ± 0.290	0.333 ± 0.098
	Degraded myelin	0.469 ± 0.149	0.969 ± 0.260	1.250 ± 0.220	**0.778 ± 0.200 (*P*= 0.05)**
Brain cells or vasculature	Neuronal cell bodies	0.189 ± 0.095	0.063 ± 0.043	0.031 ± 0.031	0.083 ± 0.047
	Astrocytic cell bodies	0.031 ± 0.031	0.031 ± 0.031	0.219 ± 0.087	0.306 ± 0.078
	Blood vessels	0.156 ± 0.065	0.094 ± 0.052	0.156 ± 0.079	0.139 ± 0.058
Myelin debris		0.500 ± 0.135	0.594 ± 0.184	0.906 ± 0.217	0.722 ± 0.181
Extracellular space		3.935 ± 0.510	2.625 ± 0.399	3.531 ± 0.557	2.750 ± 0.517
Extracellular digestion		0.438 ± 0.174	1.194 ± 0.199**	0.281 ± 0.121	0.639 ± 0.121*

*ER/Golgi*, endoplasmic reticulum and Golgi apparatus cisterna; *p.i.*, post-infection; *SEM*, standard error of the mean

Results are the mean ± SEM of 32–33 microglia per time point (6–10 microglia per animal for a total of 4 mice per time point) in the *stratum radiatum* and of 32–36 microglia per time point (7–10 microglia per animal for a total of 4 mice per time point) in the *stratum lacunosum-moleculare*

Statistical analyses were performed using a nonparametric Mann-Whitney comparison test. Results that are statistically different compared to day 0 (noninfected mice) are presented as follows: * *P* < 0.05; ** *P* < 0.01 and are indicated in bold

In the *st lac mol*, the number and alterations of microglial organelles per cell were not significantly affected during ZIKV infection (Table 4). Microglial interactions with postsynaptic dendritic spines were significantly decreased on day 7 p.i. compared with those on day 0 (*P* < 0.05) but not those with presynaptic axon terminals, which may indicate a reduction of microglia-mediated neuronal postsynaptic remodeling (Table 4; Fig. 5c,d). The interactions between microglial cells and myelinated axons, neurons, astrocytes, blood vessels, myelin debris, and extracellular space pockets were not affected during ZIKV infection. However, microglia made less contact with degraded myelin on day 7 p.i. compared with that on day 0 (*P* = 0.05) (Table 4; Fig. 5c,d). Microglial association with extracellular debris or degraded elements was also significantly higher on day 7 p.i. compared with that on day 0 (*P* < 0.05) (Table 4; Fig. 5c,d). These results suggest that microglial cells are involved in phagocytic activities, extracellular digestion, and remodeling of postsynaptic elements during ZIKV infection of the young adult mouse brain.

### Depletion of microglia affects body weight gain and survival after ZIKV challenge

**Fig. 6:**
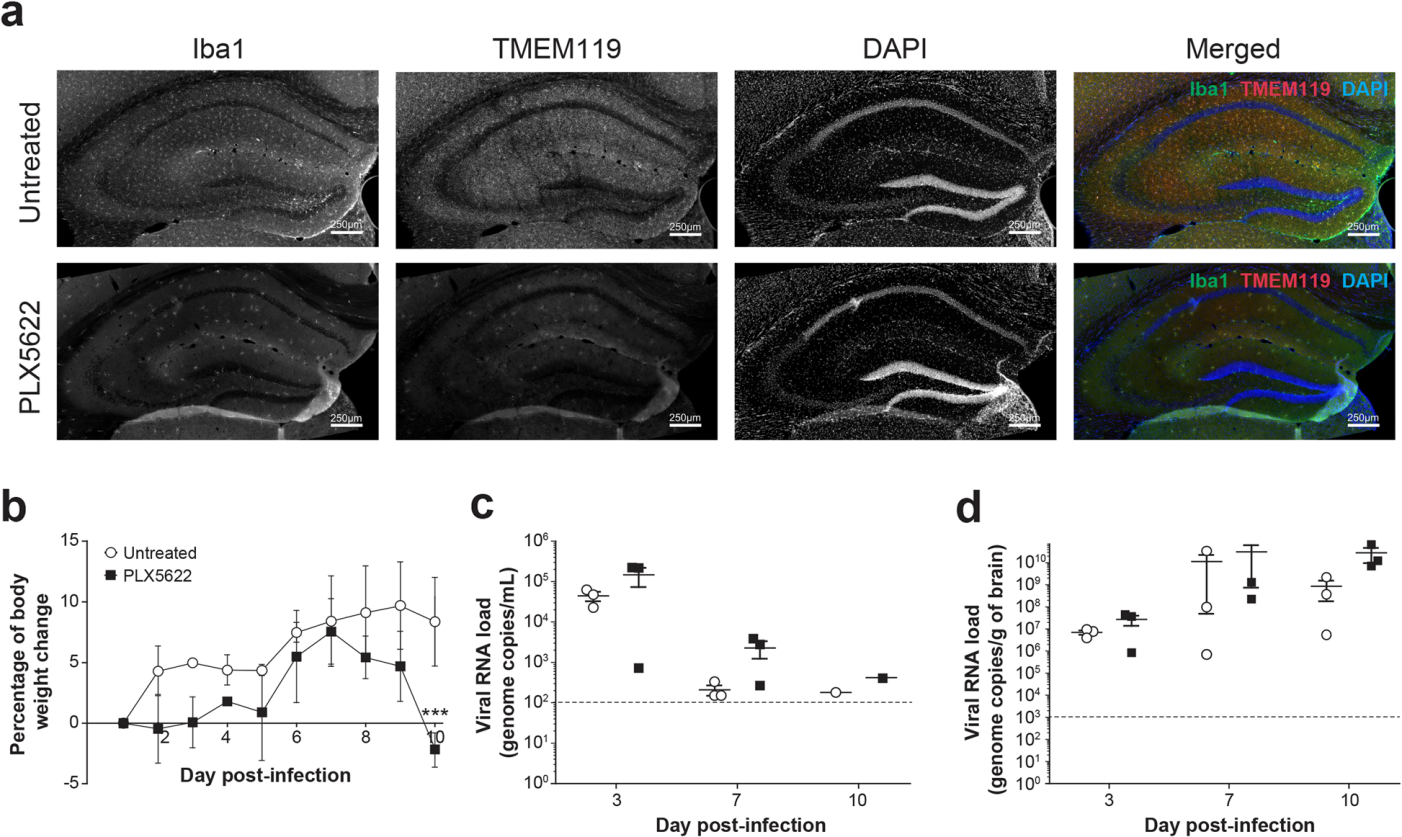
Effect of microglia depletion with PLX5622 on ZIKV infection of young adult mouse brain. **a** In a first experiment, TRIF^−/−^ × IPS-1^−/−^ mice received control or PLX5622 diet ad libitum for 10 days. Mice were sacrificed, and the brain was taken for immunofluorescence analysis. Representative micrographs illustrating the depletion of Iba1^+^/TMEM119^+^ microglia after immunofluorescence labeling in the *stratum radiatum* of untreated (upper images) and PLX5622-treated (lower images) mice. Brain sections were counterstained with DAPI. Scale bars on the picture are equivalent to 250 µm. **b–d** In a second experiment, TRIF^−/−^ × IPS-1^−/−^ mice received control or PLX5622 diet ad libitum for 10 days. Mice were then infected intravenously with 1 × 10^5^ PFUs of ZIKV strain PRVABC59 (in 100 µL volume), and the respective diets were continued until sacrifice. **b** Body weight changes of untreated (○) and PLX5622-treated (■) mice after ZIKV challenge. Results are the mean ± SEM of 3 mice per group and per time point. **c–d** Subsets of mice were sacrificed on day 0 (noninfected), 3, 7, and 10 post-infection, and viral RNA load was determined by reverse transcriptase ddPCR in serum (**c**) and brain homogenates (**d**). The dotted lines represent the limits of detection of the reverse transcriptase ddPCR assay. Results are the mean ± SEM of 3 mice per group and per time point. Statistical analyses were performed using a one-way analysis of variance with Tukey’s multiple comparison test. Results that are statistically different between indicated groups are shown as follows: *** *P* < 0.001

We first evaluated the effect of PLX5622 on the number of microglial cells and blood monocytes in TRIF^−/−^xIPS-1^−/−^ mice after 10 days of treatment. The immunofluorescence labeling of Iba1^+^/TMEM^+^ microglia was decreased in the *st rad* of the dorsal hippocampus CA1 of PLX5622-treated compared with that of untreated mice (Fig. 6a). The number of Iba1^+^/TMEM^+^ microglial cells in the *st rad* and *st lac mol* was respectively reduced by 81.9% and 79.4% after the pharmacological depletion. In contrast, the percentage of CD45^high^/CD11b^+^/Ly6C^+^ monocytes to CD45^+^ cells evaluated by flow cytometry was almost similar in treated and untreated mice (9.5 ± 1.0% *versus* 8.0 ± 0.5%) (data not shown) suggesting that PLX5622 did not affect the total number of monocytes in the blood.

In another experiment, we evaluated the effect of a microglial depletion with PLX5622 on the outcome of ZIKV infection in young adult mice. A lower increase in body weight was seen in mice treated with PLX5622 compared with that in untreated controls during the 10-day monitoring period (Fig. 6b). Furthermore, one of the 3 mice in the PLX5622-treated group died from the infection on day 10 p.i. (the brain was harvested to determine the viral RNA load), but none of the 3 control mice died. In both groups, the viremia was higher on day 3 p.i. and decreased thereafter (Fig. 6c). In untreated mice, the brain viral load reached a peak value on day 7 and decreased on day 10 p.i. (Fig. 6d). In mice treated with PLX5622, the brain viral load increased from days 3 to 7, and remained similar on day 10 p.i. The brain viral load was slightly higher in PLX5622-treated compared with that in untreated mice (2.8 × 10^10^*versus* 8.5 × 10^8^ viral genome copy numbers), but this was not statistically significant possibly due to the low number of mice per group.

### Depletion of microglia increases the number of neurons and astrocytes labeled for ZIKV antigens

**Fig. 7:**
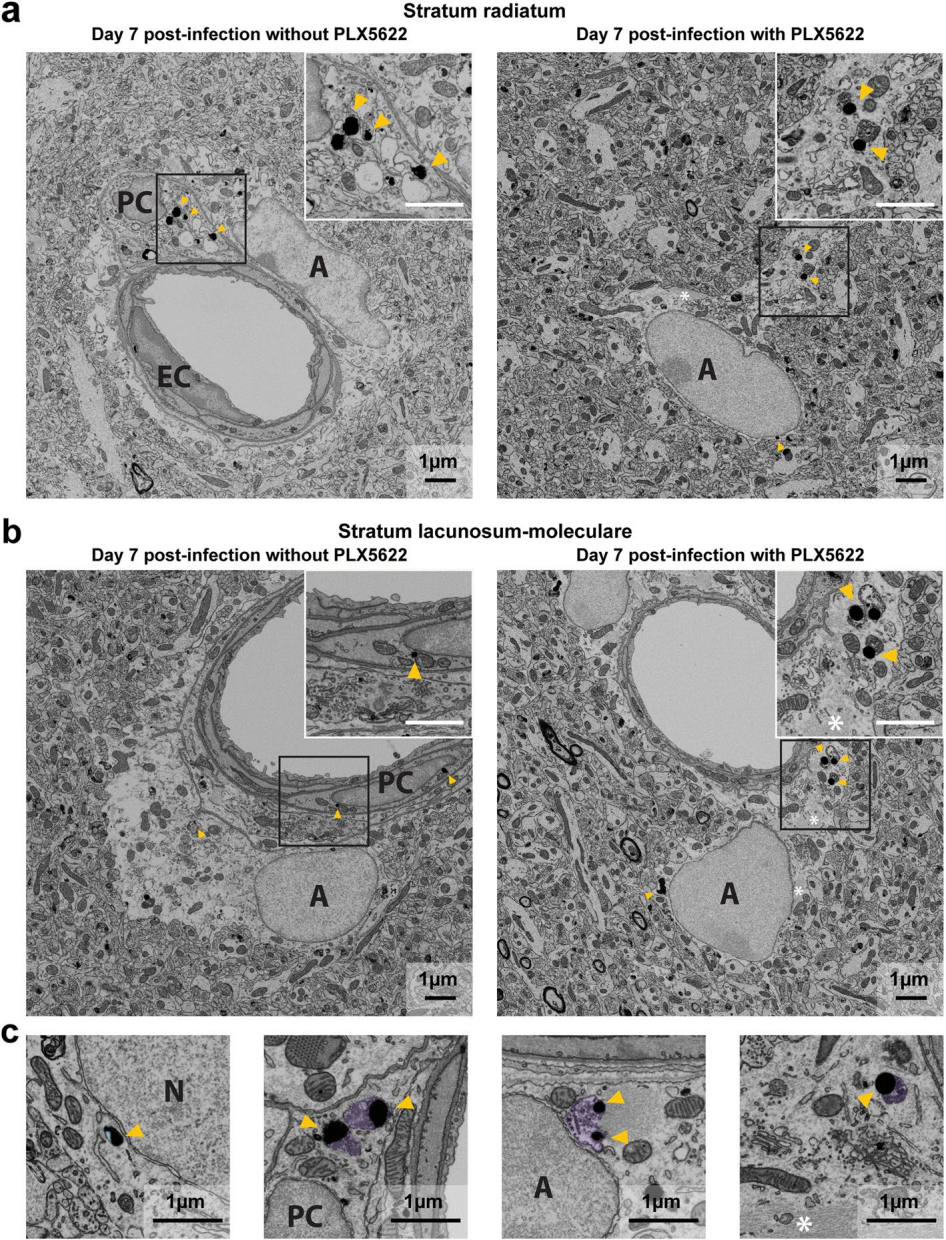
Ultrastructural analysis of the dorsal hippocampus CA1 of mice depleted in microglia and infected with ZIKV. **a** The left picture shows immunogold particles in the cytoplasm, endosome, and mitochondria of a perivascular cell in the *stratum radiatum* of untreated mice on day 7 post-infection. The right picture shows immunogold particles in the endosome of an astrocyte in the *stratum radiatum* of PLX5622-treated mice on day 7 post-infection. **b** The left picture shows nanogold particles in the mitochondria and at the nuclear membrane of a perivascular cell in the *stratum lacunosum-moleculare* of untreated mice on day 7 post-infection. The right picture shows immunogold particles in the cytoplasm and endosome of an astrocyte in the *stratum lacunosum-moleculare* of PLX5622-treated mice on day 7 post-infection. Insert in the right corner of each picture shows the subcellular localization of nanogold particles. **c** Pictures illustrate differences in content of endosomes with nanogold particles in different cell types: from left to right, neuron, perivascular cell, and astrocytes (two pictures at the right) from the *stratum lacunosum-moleculare*. Endosomes are pseudo-colored to distinguish empty endosomes (light blue) from endosomes with digested content (light purple). On the example pictures, cell types are identified by a capital letter: *A*, astrocyte; *EC*, endothelial cell, and *PC*, perivascular cell. Astrocytic intermediate filaments are also identified by a white asterisk (*). Nanogold particles, which stain ZIKV antigens, are highlighted by yellow arrowheads. Scale bars on the picture are equivalent to 1 µm for the whole picture and for the insert

In order to determine the impact of microglia on the control of viral infection, we used scanning electron microscopy to evaluate the cellular localization of ZIKV envelope antigens labeled with immunogold in the *st rad* and *st lac mol* layers of the dorsal hippocampus CA1 of mice treated or not with PLX5622. As expected from the brain viral load, the total number of cells with nanogold particles increased in the *st rad* (17 *versus* 9) and *st lac mol* (35 *versus* 21) after the pharmacological depletion of microglia compared with untreated mice (Table 5). More specifically, after microglial depletion, the percentages of neurons (58.8% in depleted *versus* 22.2% in non-depleted mice) and astrocytes (80.0% *versus* 23.8%) immunolabeled for ZIKV antigens were increased in the *st rad* and *st lac mol*, respectively. In contrast, the percentages of pericytes containing immunogold particles (0.0% *versus* 23.8%) decreased in the *st lac mol*. The percentages of other perivascular cells with nanogold particles were reduced in both layers (0.0% *versus* 22.2% in the *st rad* and 5.7% *versus* 23.8% in the *st lac mol*). Overall, the percentages of parenchymal cells with nanogold particles increased (94.1% *versus* 77.8% in the *st rad* and 91.4% *versus* 42.9% in the *st lac mol*), whereas the percentage of perivascular cells decreased (5.9% *versus* 22.2% in the *st rad* and 8.6% *versus* 57.1% in the *st lac mol*) in mice depleted in microglia suggesting a shift in the cell types with ZIKV antigens (Table 5; Fig. 7a,b). The percentages of oligodendrocytes, microglial cells, and endothelial cells containing immunogold particles remained relatively unchanged after the pharmacological depletion of microglia.

**Table 5:** Cellular localization of Zikaf virus antigens labeled with immunogold in both layers of the dorsal hippocampus CA1

Cell Types	**Number of cells with at least 2 nanogold particles (%)**			
	**Not treated with PLX5622**		**Treated with PLX5622**	
	*st rad*	*st lac mol*	*st rad*	*st lac mol*
Neurons	2 (22.2)	3 (14.3)	10 (58.8)	4 (11.4)
Astrocytes	3 (33.3)	5 (23.8)	4 (23.5)	28 (80.0)
Oligodendrocytes	0 (0.0)	0 (0.0)	1 (5.9)	0 (0.0)
Microglia	2 (22.2)	1 (4.8)	1 (5.9)	0 (0.0)
**Parenchymal cells**	**7 (77.8)**	**9 (42.9)**	**16 (94.1)**	**32 (91.4)**
Endothelial cells	0 (0.0)	2 (9.5)	0 (0.0)	1 (2.9)
Pericytes	0 (0.0)	5 (23.8)	1 (5.9)	0 (0.0)
Other perivascular cells	2 (22.2)	5 (23.8)	0 (0.0)	2 (5.7)
**Perivascular cells**	**2 (22.2)**	**12 (57.1)**	**1 (5.9)**	**3 (8.6)**
**Total cells**	**9 (100.0)**	**21 (100.0)**	**17 (100.0)**	**35 (100.0)**

% of a specific cell type to the total number of cells containing nanogold particles; *st rad, stratum radiatum*; *st lac mol*, s*tratum lacunosum-moleculare.* In the *st rad,* 36 pictures (7–13 per animal) for untreated and 47 pictures (9–14 per animal) for treated mice were analyzed. In the *st lac mol*, 51 pictures (8–16 per animal) for untreated and 59 pictures (9–22 per animal) for treated mice were analyzed. *n* = 4 mice per group

### Depletion of microglia increases viral replication in neurons and astrocytes as well as phagocytic activity of astrocytes

In order to elucidate the role of microglia in the pathogenesis of brain infection by ZIKV, we determined the subcellular localization of viral envelope antigens stained by immunogold in different brain cell types in mice treated or not with PLX5622. In untreated mice, nanogold particles were found exclusively at the plasma membrane and within endosomes of microglia, indicating their active contribution to the elimination of ZIKV-infected cells by phagocytosis (Supplementary Table 3). In PLX5622-treated mice, the number of nanogold particles was higher in the cytoplasm (9 *versus* 3 in the *st lac mol*), endoplasmic reticulum (5 *versus* 1 in the *st rad*), and endosomes (17 *versus* 2 in the *st rad* and 5 *versus* 0 in the *st lac mol*) of neurons suggesting an increased viral replication. In astrocytes, the number of nanogold particles were also higher in the cytoplasm (40 *versus* 1 in the *st lac mol*) as well as in the endosomes (10 *versus* 0 in the *st rad* and 31 *versus* 7 in the *st lac mol*) (Supplementary Table 3; Fig. 7a,b) of astrocytes suggesting enhanced viral replication. In the endosomes of neurons, nanogold particles were not associated with digested material (Fig. 7c), indicating their involvement in ZIKV entry into cells by endocytosis. In contrast, endosomes of astrocytes sometimes contained nanogold particles together with partially digested cellular debris (Fig. 7c), suggesting that these cells could partially compensate for the phagocytic elimination of ZIKV-infected cells, in the almost-complete absence of microglia. The number of nanogold particles in the Golgi apparatus, at the plasma membrane, mitochondrial membrane, nuclear membrane, and in the nucleus remained similar for all other cell types investigated in both layers of the dorsal hippocampus. Overall, these results indicate that microglia actively participate to the control of ZIKV replication and/or elimination in young adult mouse brain, as illustrated by enhanced accumulation of immunogold particles within organelles involved in viral replication. Furthermore, phagocytic elimination of ZIKV-infected cells is partly compensated by astrocytes in the almost-complete absence of the main phagocytes, microglia.

## Discussion

Contemporary ZIKV strains can invade the CNS of adult individuals following infection and are associated with a broad spectrum of neurological disorders. However, the mechanisms involved in the infection of the mature CNS are not yet elucidated. In this study, we evaluated the distribution of a contemporary ZIKV strain in the mature brain in a nonlethal immunodeficient mouse model as well as the role played by microglia during the infection. Our results demonstrate that ZIKV mostly localizes in the dorsal hippocampus region of the brain, which plays important roles in learning and memory functions [51]. In addition, microglial cells exhibit morphology characteristics of a reactive state, and display increased phagocytic activities and extracellular digestion during the infection. Pharmacological depletion of microglia amplifies the viral infection as shown by an increase in the number of neurons and astrocytes with ZIKV envelope antigens stained by immunogold in the dorsal hippocampus CA1. In neurons and astrocytes, nanogold particles are found in the compartment involved in ZIKV replication, such as cytoplasm, endoplasmic reticulum, and endosomes [52, 53]. In the absence of microglia, the phagocytic elimination of ZIKV-infected cells is partly compensated by astrocytes as indicated by the increased number of nanogold particles associated with digested debris in the endosomal compartment.

Despite a lack of clinical signs of the disease and effect on body weight, young adult TRIF^−/−^xIPS-1^−/−^ mice infected intravenously with the contemporary ZIKV strain PRVABC59 developed a viremia and a brain viral load as previously shown by our group [21]. Of note, WT C57BL/6 mice exhibited a transient viremia, and no viral load could be detected in their brain. Immunoperoxidase staining of viral envelope antigens indicated that ZIKV was distributed in several regions of the brain, and especially in the dorsal hippocampus. Several studies have already reported that ZIKV preferentially targets the hippocampus region of adult [8, 20, 30, 54, 55] and neonate mouse brains [56]. The wider distribution of ZIKV across the brain in our model compared with that seen in immunocompetent mice could result from the altered production in type I IFN as suggested by others [8, 20]. Nevertheless, our results confirm that ZIKV targets learning and memory-related regions of the brain [8] as other flaviviruses such as West Nile virus [57] and Japanese encephalitis virus [58]. In this respect, a report described the case of a ZIKV-positive adolescent presenting cognitive impairment [59].

Scanning electron microscopy analysis revealed that ZIKV envelope antigens labeled with immunogold were detected in astrocytes, neurons, pericytes, microglia as well as in other vascular cells in the *st rad* and *st lac mol* layers of the dorsal hippocampus CA1. The cell types that are infected by ZIKV in the brain widely differ according to the mouse model and route of virus inoculation used and still remain unclear. A study demonstrated that astrocytes constitute the main cell type infected by ZIKV in the brain of susceptible mice [60]. More recently, ZIKV was reported to replicate in neurons of adult human cortical tissues and mature brain of immunocompetent mice [8, 20]. Other studies also described the presence of ZIKV envelope antigens in pericytes [61] and blood vessels [62]. Finally, microglia isolated from human fetal brain and mouse brain can be infected by ZIKV and promote the release of pro-inflammatory cytokines [63–66]. We cannot exclude, however, that the lack of type I IFN secretion in our murine model could have modulated the susceptibility of the different brain cells to ZIKV infection.

ZIKV infection increases the levels of a series of cytokines (IL-1α, IL-6, IL-9, IL-10, IL-12p70, and IFN-γ) and chemokines (CCL2, CCL3, CCL4, CCL5, CCL11, and CXCL1) in mouse brain. Microglia could be the main cells responsible for producing inflammatory mediators after ZIKV infection, but it is suggested that neurons [67] and pericytes [68] could also be potential sources. ZIKV induces a pro-inflammatory response through the release of IL-6 and IL-9 as well as an anti-inflammatory response through IL-10. Astrocytes and neurons could produce CCL2 which is involved in monocyte recruitment and induces the reactivity of microglia during infection [69]. CXCL1 is involved in neutrophil migration during West Nile virus pathogenesis [70]. The release of CCL3, CCL4, and CCL5 may promote the recruitment and activation of T cells during viral infection of the brain [60]. ZIKV increases the production of IL-12p70, an inducer of IFN-γ production by T cells [71]. It is suggested that IFN-γ could signal through microglia [72] to induce cognitive dysfunction, including spatial learning defects, in adult mice that recovered from West Nile virus or ZIKV infection [55]. Finally, ZIKV promotes the release of CCL11 which was associated with a reduction of neurogenesis and cognitive functions in the hippocampus [73].

Microglia display diverse phenotypes depending on their function and interaction with their surrounding microenvironment [74]. Under normal physiological conditions, microglia adopt a surveying phenotype characterized by compact cell bodies and elongated ramification processes [25, 26]. In case of CNS injury or infection with a pathogen, microglia transform in reactive states by withdrawing or further extending their processes, and enlarging their cell bodies sometimes leading to an amoeboid morphology [29, 75]. Immunofluorescence analyses revealed that Iba1^+^/TMEM119^+^ microglial cells highly co-localize with ZIKV antigens in both layers of the dorsal hippocampus CA1. Although the density of microglia in these two regions was not affected after ZIKV challenge, microglial clustering increased in infected *versus* noninfected mice possibly as a result of innate immune activation [76]. It has been suggested that these primary microglial nodules could provide an environment for T cells leading to the formation of secondary nodules in which neurons are destroyed during viral encephalitis [77]. Furthermore, the cell body and arborization areas of microglia were significantly increased in infected *versus* noninfected mice. The enlargement of microglial cell body could result from the increased production of pro-inflammatory mediators in the brain and to the removal of cellular debris by phagocytosis or extracellular digestion, as observed in other contexts of brain injury [75, 78]. The increase in the ramification process may suggest that microglia enhance their surveillance and dynamic interaction with synaptic elements in the brain parenchyma [24, 79–81]. These characteristics are consistent with a reactivity or functional transformation of microglia during ZIKV infection as previously demonstrated in adult mouse brain [20] as well as in hippocampal slices of newborn mice [56].

Microglia have multiple functions including surveillance of the brain, elimination of superfluous synapses (pruning), as well as clearance of damaged neurons and myelin debris by phagocytosis [22, 82]. In this regard, altered microglial functions can be identified through a change in their interactions within their microenvironment at the ultrastructural level [24]. In the *st lac mol*, the interactions of microglial cells with postsynaptic dendritic spines, but not with presynaptic axon terminals, were significantly decreased on day 7 p.i. compared to noninfected mice, which may indicate a reduction of microglia-mediated neuronal postsynaptic remodeling in the active phase of infection [24]. These results may also suggest that microglia communication/interactions with postsynaptic elements is compromised, which could impact the maintenance of synaptic homeostasis following ZIKV infection of the brain [83]. Previous studies showed that T cells promote microglial elimination of postsynaptic elements by phagocytosis in the hippocampus CA3 after recovery from ZIKV infection [55]. Phagocytic inclusions or endosomes were also found in the cell bodies and processes of microglia. In the *st rad*, the number of immature lysosomes and the total number of lysosomes containing phagocytic or degraded materials were significantly increased in microglial cells of ZIKV-infected *versus* noninfected mice. These results demonstrate that microglia exhibit an enhanced capacity to phagocytose ZIKV-infected cells during brain infection. Dilation of the lumen of endoplasmic reticulum and Golgi apparatus, an ultrastructural marker of cellular stress, remained unchanged in microglia after ZIKV challenge. The number of lipidic inclusions which are actively formed in microglia responding to pro-inflammatory cytokines, and may thus represent a marker of cellular metabolic stress or an early hallmark of neuroinflammation [84], was increased in ZIKV-infected compared with that in noninfected mice, although the difference was not statistically significant. Following ZIKV challenge, microglia interacted differently with their microenvironment in the brain. Indeed, microglial cell bodies and processes were associated with an increased number of extracellular space pockets containing degraded elements or debris in both layers of the dorsal hippocampus CA1. These results suggest, for the first time to our knowledge, that microglia could play an important role in the removal of extracellular debris during ZIKV infection of the brain. It is suggested that microglia break down dead or dying neurons into pieces using exophagy before their phagocytic engulfment and digestion [46], hence allowing them to digest large debris and cells [49, 50]. During brain injury or infection with a pathogen, another important feature of microglia is their capacity to migrate to damaged regions. Microglia can release cathepsins, heparinases, and metalloproteinases to modify the extracellular matrix and promote their migration/motility during neuroinflammation [85]. In this respect, we observed a trend for a decrease in microglia-associated extracellular space after ZIKV challenge, which could indicate a decrease in microglial dynamics associated with altered physiological functions.

Depletion of microglia led to an increase in the number of brain cells with ZIKV antigens stained by immunogold, especially neurons and astrocytes, in both layers of the dorsal hippocampus CA1. The number of immunogold particles was increased in cellular compartments involved in viral replication in neurons (cytoplasm, endoplasmic reticulum, and endosomes) and astrocytes (cytoplasm and endosomes) from both regions suggesting an enhanced replication of ZIKV in these cells [52, 53]. This was reflected by a higher, although not significant, brain viral load in mice treated with PLX5622 compared with that in the untreated group on day 10 p.i. In line with our results, depletion of microglia with PLX5622 was also reported to increase the brain viral load in mouse models of infections caused by West Nile virus and Japanese encephalitis virus [86], vesicular stomatitis virus [87], mouse hepatitis virus [88] as well as in a pseudorabies virus model of herpes virus encephalitis [89]. In addition to its effect on microglial survival, it has been shown that PLX5622 may reduce B7 co-stimulatory signals on peripheral and brain antigen-presenting cells, which could limit reactivation of antiviral CD8^+^ T cells and reduce viral clearance [90].

Microglia are considered as the primary phagocytic cells in the brain, but astrocytes have also been shown to exhibit phagocytic activity after brain injury [91, 92]. Under normal physiological conditions, a dynamic cross talk between microglia and astrocytes coordinates their involvement in the phagocytosis of debris [93]. Due to their functional plasticity, astrocytes become partially responsible for debris removal in the absence of microglia [94, 95]. In our study, an increased number of nanogold particles together with digested material were detected in astrocytic endosomes suggesting that astrocytes could exert a beneficial phagocytic elimination role after microglial depletion. Overall, these results suggest that microglia are involved in the control of ZIKV replication and/or phagocytic elimination in the young adult mouse brain and that astrocytes could partially overcome the almost-complete absence of microglial cells by clearing virally infected cells by phagocytosis.

One limitation of this study is the use of mice deficient for the production of type I IFN instead of immunocompetent mice, which may have affected the distribution of ZIKV in the brain, as well as the cell types that were infected. However, these mice did not develop overt clinical signs of the disease and did not succumb to infection which allowed us to examine in detail the role played by microglia in the pathogenesis of ZIKV infection of young adult brain without the contribution of infiltrating peripheral monocytes. During the 2013–2017 epidemic, the increased number of adults presenting with neurological disorders was associated with contemporary strains of ZIKV. Nevertheless, it would be interesting to compare ZIKV strains belonging to Asian and African lineages in future studies as the latter was shown to exhibit a higher virulence in mouse models [96]. In addition, only female mice were used in our study, and a comparison with male mice could have been interesting due to the sex differences in microglia [97, 98] and immune response [99].

## Conclusions

Our results show that microglial cell bodies and processes display morphological characteristics of a reactive state or functional transformation after ZIKV infection. Microglia were found to be responsible for both the phagocytosis and extracellular digestion of degraded elements and debris. Furthermore, pharmacological depletion of microglia with PLX5622 demonstrated that these cells are normally involved in the control of ZIKV replication and/or phagocytic elimination. In addition, astrocytes could eliminate ZIKV-infected cells by phagocytosis in the absence of microglia thus revealing a partial compensatory mechanism.

## Supplementary Information

**Additional file 1.** Supplementary methods.

**Additional file 2: Supplementary Table 1. **Scoring system used to evaluate the extent of Zika virus antigen immunostaining in mouse brain.

**Additional file 3: Supplementary Table 2. **Ratios of mRNA copies of interferon-α/-β to housekeeping 18S ribosomal subunit in the brain of mice infected with Zika virus.

**Additional file 4: Supplementary Table 3. **Nanoscale subcellular localization of Zika virus antigens labeled with immunogold in both layers of the dorsal hippocampus CA1 of mice not treated or treated with PLX5622.

## Data Availability

The data that support the findings of this study are available from the corresponding authors, upon reasonable request.

## Abbreviations

ANOVA: Analysis of variance
CA1: *Cornu ammoni* 1
CCL: Chemokine (C–C motif) ligand
CD: Cluster of differentiation
CNS: Central nervous system
CSF: Cerebrospinal fluid
CXCL: Chemokine (CXC motif) ligand
DAB: Diaminobenzidine
ddPCR: Droplet digital PCR
G-CSF: Granulocyte colony-stimulating factor
Iba1: Ionized calcium-binding adaptor molecule 1
IFN: Interferon
IFNAR: Interferon alpha/beta receptor
IL: Interleukin
IPS-1: IFN-β promoter stimulator 1
Ly6C: Lymphocyte antigen 6 complex
MDA-5: Melanoma differentiation-associated gene 5
mRNA: Messenger RNA
NND: Nearest neighbor distance
NS5: Nonstructural protein 5
PB: Phosphate buffer
PBS: Phosphate-buffered saline
PFA: Paraformaldehyde
PFU: Plaque-forming units
p.i.: Post-infection
RIG-I: Retinoic acid–inducible gene I
RT: Room temperature
SEM: Standard error of the mean
STAT2: Signal transducer and activator of transcription 2
*st lac mol*: *Stratum lacunosum-moleculare*
*st rad*: *Stratum radiatum*
TBS: Tris-buffered saline
TBS-T: Tris-buffered saline containing 0.05% Triton X-100
TLR3: Toll-like receptor 3
TMEM119: Transmembrane protein 119
TNF-α: Tumor necrosis factor-α
TRIF: Toll-interleukin-1 receptor domain-containing adaptor inducing IFN-β
WT: Wild type
ZIKV: Zika virus
