# Supplementary material for: Microglia are involved in phagocytosis and extracellular digestion during Zika virus encephalitis in young adult immunodeficient mice
Source: J Neuroinflammation. 2021 Aug 16;18:178. doi: 10.1186/s12974-021-02221-z (PMC8369691; doi:10.1186/s12974-021-02221-z)
Supplement: Supplementary file 1 — Additional file 1. Supplementary methods. [file 12974_2021_2221_MOESM1_ESM.pdf]

Supplementary methods

Quantification of interferon-α/-β mRNAs by reverse transcriptase droplet digital PCR.

Total RNA was extracted from 1 mL of Trizol/brain homogenate mixtures (900 µL: 100 µL) using Direct-zol RNA MiniPrep Plus Kit (Zymo Research Corporation, Irvine, CA) as described in the manufacturer's instructions. Total RNA concentrations in the extracts, measured using a NanoDrop™ One Microvolume Spectrophotometer (Thermo Fisher Scientific, Hampton, NH), were adjusted to 80 ng per total reaction mix (30 µL) to generate cDNAs using random hexamers and the SuperScript™ IV polymerase (both from Invitrogen, Carlsbad, CA). Reverse transcriptase reaction was performed as described in the manufacturer's instructions. For the PCR step, a volume of 5 µL of cDNAs was added to 20 µL of reaction mix (QX200™ ddPCR™ EvaGreen® Supermix; Bio-Rad Laboratories, Mississauga, ON, Canada) containing primers specific to interferon (IFN)-α and IFN-β genes as well as the 18S housekeeping gene (available upon request). PCR reactions were performed in droplets produced with the QX200 droplet generator (Bio-Rad Laboratories). Droplet-partitioned samples were then transferred to a 96-well plate, sealed, and cycled in a C1000 deep-well thermocycler (Bio-Rad Laboratories) under the following cycling protocol: 95ºC for 5 min (DNA polymerase activation), 40 cycles at 95ºC for 30 s and 57.8ºC for 1 min, post-cycling steps at 4ºC for 5 min and then at 98ºC for 10 min (enzyme inactivation) and an infinite hold at 12ºC. The plate was then read in the FAM channel of the QX200 droplet reader (Bio-Rad Laboratories). Data were analyzed using the QuantaSoft software (Version 1.7.4; Bio-Rad Laboratories). The numbers of mRNA

copies of IFN-α/-β were normalized to those of the housekeeping 18S ribosomal subunit in each sample.

Determination of blood monocyte levels by flow cytometry analysis.

Blood samples (~150 µL) were collected from the mandibular vein of mice and kept in EDTA-coated tubes (ThermoFisher Scientific, Waltham, MA) to prevent coagulation. A volume of 100 µL of blood sample was incubated with 5 mL of Red Blood Cell Lysis Buffer 1× (BioLegend, San Diego, CA) at room tempearture for 20 min. Cell suspensions (100 µL) were washed once with Dulbecco's PBS (DPBS) and incubated with purified rat anti-mouse CD16/CD32 antibody (1:100) (clone 2.4G2; BD Biosciences, San Jose, CA) on ice for 30 min to block non-specific binding. After a single washing step with DPBS, cell suspensions (100 µL) were labeled with APC-Cy7-CD45 (clone 30-F11; BD Biosciences), BUV737-CD11b (clone M1/70; BD Bioscience) and BV605-Ly6C (clone AL21; BD Biosciences) rat anti-mouse antibodies at 4°C for 40 min. Flow cytometry analyses and data acquisition were performed using a BD SORP LSR II and the BD FACSDiva software (both from BD Biosciences), respectively.
