# Supplementary material for: Microglia are involved in phagocytosis and extracellular digestion during Zika virus encephalitis in young adult immunodeficient mice
Source: J Neuroinflammation. 2021 Aug 16;18:178. doi: 10.1186/s12974-021-02221-z (PMC8369691; doi:10.1186/s12974-021-02221-z)
Supplement: Supplementary file 2 — Additional file 2: Supplementary Table 1. Scoring system used to evaluate the extent of Zika virus antigen immunostaining in mouse brain. [file 12974_2021_2221_MOESM2_ESM.pdf]

Supplementary Table 1. Scoring system used to evaluate the extent of Zika virus antigen immunostaining in mouse brain.

**Score**	**Definition**
Grade 0	No or very weak staining.
Grade 1	Weak staining partially covering the analyzed region.
Grade 2	Moderate staining covering partially or almost entirely the analyzed region; weak staining covering entirely the analyzed region.
Grade 3	Moderate staining covering almost entirely or entirely the analyzed region or intense staining partially covering the analyzed region.
Grade 4	Intense staining covering almost entirely the analyzed region or moderate to intense staining covering entirely the analyzed region.
Grade 5	Intense staining covering entirely the analyzed region.
