# Supplementary material for: Microglia are involved in phagocytosis and extracellular digestion during Zika virus encephalitis in young adult immunodeficient mice
Source: J Neuroinflammation. 2021 Aug 16;18:178. doi: 10.1186/s12974-021-02221-z (PMC8369691; doi:10.1186/s12974-021-02221-z)
Supplement: Supplementary file 3 — Additional file 3: Supplementary Table 2. Ratios of mRNA copies of interferon-α/-β to housekeeping 18S ribosomal subunit in the brain of mice infected with Zika virus. [file 12974_2021_2221_MOESM3_ESM.pdf]

Supplementary Table 2. Ratios of mRNA copies of interferon-α/-β to housekeeping 18S ribosomal subunit in the brain of mice infected with Zika virus.

**Day post-infection**	**Ratio IFN-α to 18S mRNAs**<br>**Mean ± SEM (a.u.)**	**Ratio IFN-β to 18S mRNAs**<br>**Mean ± SEM (a.u.)**
0 (non-infected)	$6.14x10^{-7}±1.86x10^{-7}$	$2.18x10^{-8}±6.22x10^{-9}$
3	$6.89x10^{-7}±1.53x10^{-7}$	$2.63x10^{-8}±5.85x10^{-9}$
7	$6.45x10^{-7}±1.47x10^{-7}$	$4.15x10^{-8}±1.04x10^{-8}$
10	$4.58x10^{-7}±3.46x10^{-8}$	$1.68x10^{-8}±1.51x10^{-9}$
14	$4.50x10^{-7}±2.18x10^{-8}$	$2.78x10^{-8}±4.20x10^{-9}$

a.u., arbitrary unit; SEM, standard error of the mean.
Results are the mean ± SEM of 5-6 mice per time point.
