# Supplementary material for: Microglia are involved in phagocytosis and extracellular digestion during Zika virus encephalitis in young adult immunodeficient mice
Source: J Neuroinflammation. 2021 Aug 16;18:178. doi: 10.1186/s12974-021-02221-z (PMC8369691; doi:10.1186/s12974-021-02221-z)
Supplement: Supplementary file 4 — Additional file 4: Supplementary Table 3. Nanoscale subcellular localization of Zika virus antigens labeled with immunogold in both layers of the dorsal hippocampus CA1 of mice not treated or treated with PLX5622. [file 12974_2021_2221_MOESM4_ESM.pdf]

Supplementary Table 3. Nanoscale subcellular localization of Zika virus antigens labeled with immunogold in both layers of the dorsal hippocampus CA1 of mice not treated or treated with PLX5622.

**Parameters**		**Number of nanogold particles**						
		Parenchymal cells				Vascular cells		
		Neuron	Astro	Oligo	Microglia	EC	Pericyte	Other
**Not treated with PLX5622**								
st rad	Cytoplasm	1	2	0	0	0	0	1
	Endosome	2	0	0	3	0	0	2
	Endoplasmic reticulum	1	1	0	0	0	0	0
	Golgi	0	0	0	0	0	0	0
	Plasma membrane	0	1	0	2	0	0	1
	Mitochondrial membrane	0	0	0	0	0	0	0
	Nuclear membrane	1	2	0	0	0	0	1
	Nucleus	0	1	0	0	0	0	0
	**Total**	**5**	**7**	**0**	**5**	**0**	**0**	**5**
st lac mol	Cytoplasm	3	1	0	0	0	2	5
	Endosome	0	7	0	6	1	2	1
	Endoplasmic reticulum	1	4	0	0	0	3	3
	Golgi	0	0	0	0	0	1	0
	Plasma membrane	1	0	0	0	1	2	0
	Mitochondrial membrane	0	0	0	0	0	0	0
	Nuclear membrane	2	0	0	0	1	2	4
	Nucleus	0	6	0	0	0	2	0
	**Total**	**7**	**18**	**0**	**6**	**3**	**14**	**13**
**Treated with PLX5622**								
st rad	Cytoplasm	9	3	0	0	0	0	0
	Endosome	17	10	0	0	0	1	0
	Endoplasmic reticulum	5	1	1	2	0	0	0
	Golgi	0	0	0	0	0	0	0
	Plasma membrane	0	0	0	0	0	0	0
	Mitochondrial membrane	0	0	0	0	0	0	0
	Nuclear membrane	2	1	5	0	0	1	0
	Nucleus	0	0	0	0	0	0	0
	**Total**	**33**	**15**	**6**	**2**	**0**	**2**	**0**
st lac mol	Cytoplasm	9	40	0	0	0	0	0
	Endosome	5	31	0	0	2	0	5
	Endoplasmic reticulum	0	9	0	0	0	0	0
	Golgi	0	0	0	0	0	0	0
	Plasma membrane	1	3	0	0	0	0	0
	Mitochondrial membrane	0	0	0	0	0	0	0

	Nuclear membrane	2	13	0	0	0	0	1
	Nucleus	0	1	0	0	0	0	0
	**Total**	**17**	**97**	**0**	**0**	**2**	**0**	**6**

Astro, astrocyte; EC, endothelial cell; Oligo, oligodendrocyte; *st rad, stratum radiatum*; *st lac mol, stratum lacunosum-moleculare*. The subcellular localization of nanogold particles was determined in the different cells reported in the Table 5 of the manuscript.
